# Pesticide Residue Fast Screening Using Thermal Desorption Multi-Scheme Chemical Ionization Mass Spectrometry (TD-MION MS) with Selective Chemical Ionization

**DOI:** 10.1021/acsomega.3c00385

**Published:** 2023-07-15

**Authors:** Fariba Partovi, Joona Mikkilä, Siddharth Iyer, Jyri Mikkilä, Jussi Kontro, Suvi Ojanperä, Paxton Juuti, Juha Kangasluoma, Aleksei Shcherbinin, Matti Rissanen

**Affiliations:** † Karsa Ltd., A. I. Virtasen aukio 1, Helsinki 00560, Finland; ‡ Aerosol Physics Laboratory, Physics Unit, Faculty of Engineering and Natural Sciences, Tampere University, Tampere 33720, Finland; § Finnish Customs, P.O. Box 512, Helsinki FI-00101, Finland; ∥ Institute for Atmospheric and Earth System Research/Physics, Faculty of Science, University of Helsinki, Helsinki 00014, Finland; ⊥ Department of Chemistry, University of Helsinki, Helsinki 00014, Finland

## Abstract

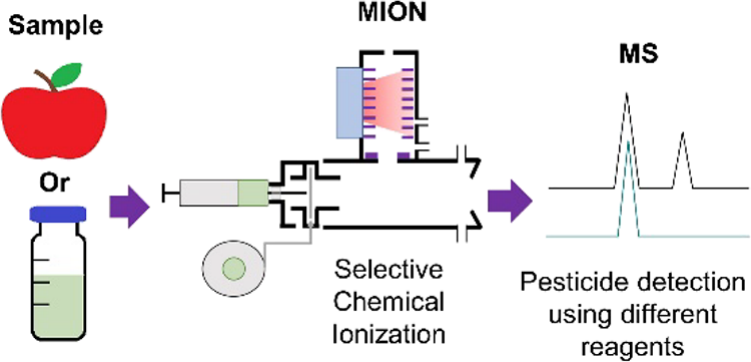

In this work, the detection characteristics of a large group of common pesticides were investigated using a multi-scheme chemical ionization inlet (MION) with a thermal desorption unit (Karsa Ltd.) connected to an Orbitrap (Velos Pro, Thermo Fisher Scientific) mass spectrometer. Standard pesticide mixtures, fruit extracts, untreated fruit juice, and whole fruit samples were inspected. The pesticide mixtures contained 1 ng of each individual target. Altogether, 115 pesticides were detected, with a set of different reagents (i.e., dibromomethane, acetonylacetone, and water) in different polarity modes. The measurement methodology presented was developed to minimize the common bottlenecks originating from sample pretreatments and nonetheless was able to retrieve 92% of the most common pesticides regularly analyzed with standardized UHPLC–MSMS (ultra-high-performance liquid chromatography with tandem mass spectrometry) procedures. The fraction of detected targets of two standard pesticide mixtures generally quantified by GC–MSMS (gas chromatography with tandem mass spectrometry) methodology was much less, equaling 45 and 34%. The pineapple swabbing experiment led to the detection of fludioxonil and diazinon below their respective maximum residue levels (MRLs), whereas measurements of untreated pineapple juice and other fruit extracts led to retrieval of dimethomorph, dinotefuran, imazalil, azoxystrobin, thiabendazole, fludioxonil, and diazinon, also below their MRL. The potential for mutual detection was investigated by mixing two standard solutions and by spiking an extract of fruit with a pesticide’s solution, and subsequently, individual compounds were simultaneously detected. For a selected subgroup of compounds, the bromide (Br^–^) chemical ionization characteristics were further inspected using quantum chemical computations to illustrate the structural features leading to their sensitive detection. Importantly, pesticides could be detected in actual extract and fruit samples, which demonstrates the potential of our fast screening method.

## Introduction

According to the World Health Organization (WHO), pesticides are substances used to prevent, control, or destroy pests, diseases, and weeds in plants, as well as to protect humans from vector-borne diseases.^1,2^ They are classified into different types based on their purpose, such as herbicides, insecticides, and fungicides.^3^ Pesticides play a vital role in modern agriculture as they help mitigate significant losses in crop production caused by pests and diseases. Their usage has steadily increased over the years, reaching approximately 4.1 million tons annually.^4,5^ With the world’s population projected to reach 9.7 billion by 2050, the demand for highly productive crops is expected to rise, making pesticides essential.^6^ However, it is important to recognize that pesticides can be toxic and pose both acute and chronic health risks, depending on exposure levels and methods.^7^

International conventions provide the means for countries to protect their populations from exposure to toxic compounds. However, pesticides are used in large quantities, which leads to potential health risks for the consumers. A maximum residue level (MRL) is the highest level of residue that is legally tolerated, and it is distinct for each pesticide and for each sample type (e.g., fludioxonil’s MRL in pineapple is 7 mg/kg, while that of diazinon for the same fruit is 0.3 mg/kg).^8^ Considerable progress has been made in the development of fast screening techniques to monitor pesticide levels in food, including the paper spray ionization mass spectrometer (PSI-MS)^9^ and desorption electrospray ionization (DESI).^10^ While some methods may not meet the desired speed, there are several effective and efficient techniques currently available for this purpose. It is important to note, however, that only a small proportion of all food products are subject to testing using standardized methods. Some of the most common in use are GC–MS/MS (gas chromatography with tandem mass spectrometry) and LC–MS/MS (liquid chromatography with tandem mass spectrometry), which generally require complicated sample preparation.^11^ Nonetheless, SERS (surface-enhanced Raman spectroscopy) and TD-ESI/MS/MS (thermal desorption electrospray ionization tandem mass spectrometry) are reported as methods for rapid detection of pesticide residues on surfaces of fruits and vegetables.^12^ The drawback of such methodology is that they do not include various sample matrices but are limited on the surfaces of the goods, leaving the potentially imbedded toxins unresolved.

Chemical ionization mass spectrometry (CIMS) is a form of mass spectrometry wherein the ionization of a substance is accomplished by reactions with a set of ions, which serve as ionizing reagents.^13^ The technique was introduced by Munson and Field in 1966,^14^ and since then it has been applied in numerous branches of chemistry and biochemistry. In principle, it is possible to selectively ionize any component of interest in the matrix by a suitable chemical ionization reaction.^15^ With the right selection of reagent ions, CIMS can offer a soft, selective, and extremely sensitive online detection of virtually any gas-phase compound, and this feature was pursued here.^16^

Thermal desorption multi-scheme chemical ionization (TD-MION) is an advanced analytical technique that combines thermal desorption with chemical ionization to detect and quantify compounds in various matrices. The TD-MION technique employs a multi-scheme chemical ionization source that selectively ionizes molecules released from the thermal desorption system. The ionized molecules are then detected and quantified using a mass spectrometer. In the current work, the capabilities are extended by the novel high-throughput, atmospheric pressure multi-scheme ionization inlet, which allows to switch between ion chemistries in both polarities.

Both APCI (atmospheric pressure chemical ionization),^17^ a commonly used ionization technique in mass spectrometry, and the MION inlet operate under atmospheric pressure. However, they differ in their principles of chemical ionization. In APCI, analytes are converted into droplets through a nebulizer and undergo desolvation and ionization via a corona discharge. In contrast, MION uses X-ray radiation to generate reagent ions, which are accelerated and directed toward the sample flow for chemical ionization of target pesticides. MION’s ion guns, which consist of metal ring electrodes forming electric field funnels, incorporate purge flows that allow only the charged reagent molecules to mix with the sample flow. This facilitates selective interaction between the reagent ions and the target compounds.

In the current work, the CIMS detection sensitivity as a function of the reagent ion and the structure of the target pesticide were investigated. With a large collection of different pesticides, the sensitivity and selectivity of the method could be examined. As a proof of concept, several modes of sample introduction were inspected, including sample extract injection, untreated juice injection, and swabbing of a filter against the surface of a fruit. Additionally, the bromide (Br−) chemical ionization was further investigated by quantum chemical computations for a selected subgroup of compounds. The computed stabilities of the bromide-pesticide adducts were compared to the experimental detection sensitivities, which can be used for a rapid prediction of favorable reagent ion–target molecule combinations.

## Experimental Section

### Materials

**Figure 1:**
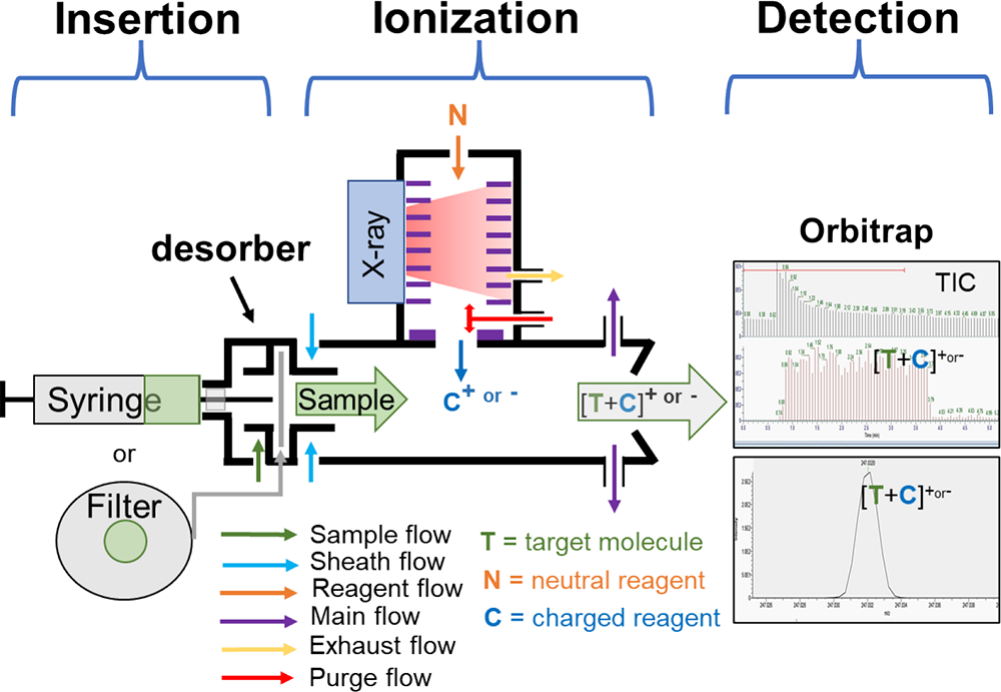
Schematic presentation of the thermal desorption unit and the MION ion source (i.e., TD-MION).

Experiments were conducted at the laboratory of Karsa Ltd., Helsinki, applying MION coupled to an upgraded LTQ Velos Pro (Linear Trap Quadrupole) Orbitrap mass spectrometer (Thermo Fisher, mass resolving power up to 100,000). A custom-made thermal desorption unit (Karsa Ltd.) was mounted upstream of the MION, comprising a filter holder and an injection port constituted in a TD-MION system. MION utilizes several parallel ion schemes, which can be operated in rapid succession in a consecutive manner.^16^ MION’s ion guns, which consist of electric field funnels made of metal ring electrodes, contain purge flows and thereby allow exclusively the charged reagent molecules to mix with the sample flow. Rapid selection of the reagent ion is realized by switching of electric fields, and the current MION design accommodates three different ion sources at a constant reaction time.^18^ Combined with fast polarity switching, it generates six unique ionization schemes and makes the combination of MION and Orbitrap mass spectrometry a powerful and chemically selective detection method. While the capability of switching between reagent ions has been demonstrated previously,^19−21^ the ionization in these studies was carried out in low pressure, whereas the MION source operates at atmospheric pressure. A schematic of the experimental setup of the TD-MION-Orbitrap mass spectrometry used in the current work is shown in Figure 1.

Dibromomethane (DBrMe; CH_2_Br_2_) and 2,5-hexanedione (acetonylacetone, Acac; [(CH_3_C(*O*)CH_2_)_2_]) were used as reagents for producing bromide (Br^–^) and protonated Acac (C_6_H_10_O_2_H^+^) ions, respectively (Table 1). When only an air feed, scrubbed and filtered from organic and inorganic residues using a Purafil Charcoal scrubber (Ecotech, USA), was used in the ion source, the trace amounts of water present led to the production of H_3_O^+^, which was used for the proton transfer reactions. Similarly, the O_2_ in the dopant free air feed is a source of  and was used for negative polarity ionization. Although no dopants were added in these cases, other trace impurities present in the feed could perceivably contribute to the ionization reactions. The reagent ions were generated by X-ray radiation (4.9 keV Hamamatsu L12536), accelerated in the electric funnels, and shot to the center of the sample flow, leading to chemical ionization of the target pesticides. The targets were then detected as protonated or deprotonated molecules, depending on the used polarity, or as an adduct when a reagent supply was used (i.e., Acac or DBrMe). At each level of the study, we fed the chemical ionization inlet with different reagent solutions (Table 1). Separate reagent feed towers were used for each reagent.

**Table 1:**
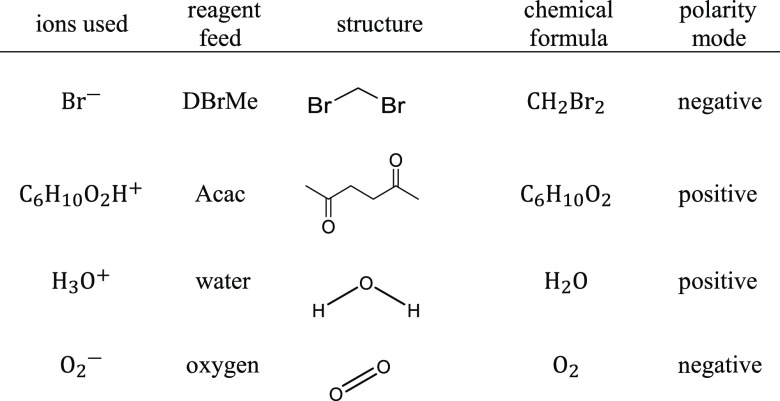
Reagent Ion Chemistries Used in the Current Work

The pesticide standard mixtures, sourced from LGC Standards Ltd. (UK), underwent dilution in acetonitrile by the Finnish Customs. Subsequently, the prepared solutions were handed over to us, enabling us to proceed with our analysis. The LC pesticide mixture comprised 73 pesticides, while the two GC pesticide mixtures (GC1 and GC2) contained 65 and 70 pesticides, respectively. These were combined into two mixtures: mixture “A” consisted of LC and GC1, totaling 138 different pesticides with a concentration of 1 ng/μL each, while mixture “B” solely comprised GC2 with 70 different pesticides, having a lower concentration of approximately 0.3 ng/μL per sample. The “LC” and “GC” in the names denoted their respective analysis methods, i.e., liquid chromatography–mass spectrometry (LC–MS) and gas chromatography–mass spectrometry (GC–MS). Real fruit extracts were homogenized using a Retsch GM300 grinder with dry ice, and 10 g of homogenate was extracted with 10 mL of acetonitrile. A salting-out mixture consisting of 1 g of sodium chloride, 1 g of sodium citrate, and 0.5 g sodium hydrogen acetate was added. After shaking and centrifugation, the extract was purified using PSA and MgSO_4_. Following centrifugation, the extract was filtered into a glass vial. Sample preparation and analysis were performed in accordance with the CEN EN 15662 standard. The real fruit extracts and samples (i.e., sliced fruits) were provided by the Finnish Customs. The list of all the pesticides investigated is available in the Supporting Information (Table S1 and Table S2). Syringe injections were performed by a 10 μL Trajan Scientific and Medical SGE 10FX-5C syringe. Custom-made filters (metal mesh filter, 37 mm diameter, Karsa Ltd.) were used for filter desorption measurements of the standard mixtures and fruit extracts. Similar filters were employed for swabbing the whole fruit samples.

### Methods

Standard solution measurements were carried out by injecting the solutions with 1 ng of each pesticide directly onto a filter for a subsequent thermal desorption. Acetone was used as the blank sample and as the washing solution. Fruit extract measurements were performed by injecting 2 μL of fruit extract directly into the system, with acetonitrile used as the blank solution, as well as for washing. In addition to the previously described sampling method, a swabbing technique was implemented to collect the sample. This involved utilizing a custom-made filter, manufactured by Karsa Ltd., composed of a metal mesh with a diameter of 37 mm. The filter was swabbed across the non-defined surface of the sliced fruit to obtain the sample. Following this step, the filter was placed in the desorber to prepare it for further analysis. In this study, a pineapple was used for this purpose. After the swab-sampling was completed, the filter was placed in the desorbing unit’s filter holder and then thermally desorbed. The untreated juice exuded from the same sample (pineapple slices) was also subjected to investigation. This measurement was performed by injecting 5 μL of the juice on the filter, placing it in the filter holder assembly, and subsequently thermally desorbing it.

For all the measurements, a temperature ramping was performed from 30 to 250 °C in 90 s. Then, the temperature was kept at 250 °C for another 450 s. The total ion current (TIC) and the spectra of the target were both recorded for approximately 540 s. During this time, the sample evaporated and passed to the inlet guided by gas flows. Data was collected using the 100–880 *m*/*z* (mass-to-charge ratio) mass range (full-scan mode, 100 k resolution, three microscans). Data analysis was performed using TraceFinder General Quan 4.1 software (Thermo Fisher).

The integrated desorption profile peak area (PA) was used as the measure of detection efficiency. It is the target signal obtained during the whole desorption time and thus presents the cumulative signal obtained from the target during a single experiment (see SI Figure S1 for a graphical explanation). We chose this measure of the detection efficiency as the compounds desorb with different efficacy and in different timescales, and the chosen methodology aims to account for this behavior.

### Molecular Modeling Quantum Chemical Computation

The usefulness of bromide ionization for detecting a variety of pesticides was assessed by computing bromide binding to the target molecules. A subgroup of carefully selected pesticides was chosen, representing various common chemical structures present in these compounds (Supporting Information Table S3). A systematic conformer sampling was carried out on the free molecules and adducts with the Spartan ‘20 program^16^ using the molecular mechanics force field (MMFF) method. Single-point energies were calculated on all conformers using density functional theory (DFT) with the B3LYP/6-31+G(d) method, followed by a geometry optimization step at the same level of theory on conformers within 5 kcal/mol of the lowest energy conformer. These calculations were performed using the Gaussian ‘16 program.^20^ Adduct formation enthalpies (Δ*H*) were calculated by subtracting the enthalpy of the lowest energy adduct geometry from that of the lowest-energy free molecule and ion. In order to estimate an error margin for the B3LYP/6-31+G(d) calculated formation enthalpies, the electronic energies of the adduct, free molecule, and ion were recalculated at the more accurate DLPNO-CCSD(T)/aug-cc-pVTZ-PP level of theory for a subset of five pesticides using the ORCA 4.2.1 program.^22^ A maximum difference of 2.6 kcal/mol is observed, with the B3LYP/6-31+G(d) method showing a proclivity to underestimate adduct formation enthalpies (see Table S6 in the Supporting Information).

While the conformer sampling algorithm in Spartan ‘20 should reliably find the lowest energy bromide adduct ([M + Br]^−^) conformers for analytes with strong hydrogen-bond-donating functional groups,^23^ its reliability could be less certain for analytes without these functional groups, such as most of the pesticide molecules considered in this study (fludioxonil, linuron, prometryn, etc.). In these cases, the conformer list was checked by eye to ensure that all possible Br^–^ ion positions around the analyte were considered for the subsequent DFT steps.

## Results and Discussion

The pesticide mixtures and fruit samples were measured with the selected ion schemes. In the negative polarity spectra, the targets of the data analysis were the pesticide adducts with bromide ([M + Br]^−^) and the deprotonated molecules ([M – H]^−^), whereas in the positive polarity spectra, the targets were the protonated Acac adducts ([M + Acac + H]^+^) and protonated ([M + H]^+^) pesticide ions, respectively. Altogether, with all the reagents in different modes, 115 pesticides out of 208 were detected. This represents 56% of all the targets present in the mixtures, including several of the most used pesticides. The number of the detected compounds from each mixture and with each ionization method is illustrated in Table 2. Details related to detection of individual pesticides with different reagents is presented in Supporting Information Tables S1 and S2. The confidence of the detection is separated into two categories, “confirmed detection” and “detected”, based on the match between expected and observed isotopic patterns.^23^ The mismatch between the isotopes of the target and the observed amount is due to the way Orbitrap data is processed, as only signals above a certain threshold appear in the spectrum and the smaller signals are being removed from the spectra.

**Table 2:** Numbers of Detected Pesticides from Each Pesticide Mixture and the Reagent Ion Chemistries Used

		“A” mixture		“B” mixture
		“LC” mix	“GC1” mix	“GC2” mix
total number of species		73	65	70

		*confirmed detection*	*detected*	*not detected*	*confirmed detection*	*detected*	*not detected*	*confirmed detection*	*detected*	*not detected*
Reagent	Adduct	67	4	2	29	11	25	24	24	22
DBrMe	[M+Br]–	25	7	41	11	4	50	6	16	48
DBrMe	[M–H]–	4	4	65	3	5	57	2	10	58
Acac	[M+Acac]+	2	13	58	0	4	61	0	0	70
Acac	[M+H]+	57	8	8	19	6	40	21	13	36
H_3_O^+^	[M+H]+	31	35	7	12	15	38	2	26	42
	[M–H]–	23	5	45	7	5	53	3	7	60

The LC pesticide mixture contained the most detected compounds with 67 confirmed detections out of a mixture of 73 pesticides. This leads to a detection of 92%, which increases to 97% when the “detected” category with mismatch in the retrieved isotopes is included in the results. The GC1 and GC2 mixtures were detected with 45 and 34%, respectively. It is interesting to note that the compounds commonly determined by LC–MS analysis are better detected than the compounds routinely analyzed with GC–MS, as one could expect that the used thermal desorption analysis is closer to a GC than an LC detection methodology. Yet, the ionization in LC–MS techniques is typically performed using electrospray ion sources, which are more similar to the atmospheric pressure chemical ionization employed in this study than the ionization methods commonly used in GC–MS, such as electron impact and the flame ionization detector.

The LC and GC1 pesticide mixtures were injected simultaneously to examine their potential influence on the results. Similar results were gained as when injecting them individually. The results suggest that at least at these target concentrations, the existence of other targets does not affect the chemical ionization quantification of the individual pesticides.

Actual fruit slices and fruit extracts were also investigated. Fruit extracts were prepared by the QuEChERS (Quick, Easy, Cheap, Effective, Rugged, and Safe) method, which consists of several steps (i.e., homogenization, adding solvent, liquid extraction, buffering and drying etc.).^24^ These same samples were previously quantified by the Finnish Customs laboratory (Finnish Accreditation Service FINAS T006, accreditation requirements SFS-EN ISO/IEC 17025) using UHPLC–MS/MS (Waters TQ-XS UHPLC–MS/MS) or GC–MS/MS (Agilent 7010B GC–MS/MS), providing the quantitative concentrations used to validate the obtained results (Table 3). Extracts of watermelon, lime, avocado, and pineapple were investigated.

**Table 3:** The Integrated Thermal Desorption Profile Peak Areas (PAs) of Detected Pesticides in Different Sample Matrices and the Corresponding Quantitative Results Gained with Validated Methods

	Formula	adduct	peak area	quantitative detection (mg/kg)^a^
**Watermelon extract**				
dimethomorph	C_21_H_22_ClNO_4_	[M+H]^+^	8.79E2	0.010
dinotefuran	C_7_H_15_N_4_O_3_^+^	[M+Br]^−^	2.41E3	0.011
imazalil	C_14_H_14_N_2_Cl_2_O	[M+H]^+^	1.12E4	0.83
**Lime extract**				
azoxystrobin	C_22_H_17_N_3_O_5_	[M+H]^+^	1.74E3	0.022
imazalil	C_14_H_14_N_2_Cl_2_O	[M+H]^+^	1.09E5	1.5
**Avocado extract**				
thiabendazole	C_10_H_7_N_3_S	[M+H]^+^	1.57E5	0.51
**Pineapple extract**				
fludioxonil	C_12_H_6_F_2_N_2_O_2_	[M+Br]^−^	5.35E5	0.48
diazinon^b^	C_12_H_21_N_2_O_3_PS	[M+H]^+^	1.1E3	0.011
**Pineapple swabbing**^c^				
fludioxonil	C_12_H_6_F_2_N_2_O_2_	[M+Br]^−^	4.2E5	0.48
diazinon	C_12_H_21_N_2_O_3_PS	[M+H]^+^	7.9E2	0.011
**Pineapple juice**				
fludioxonil	C_12_H_6_F_2_N_2_O_2_	[M+Br]^−^	4.4E3	0.48
diazinon	C_12_H_21_N_2_O_3_PS	[M+H]^+^	1.1E2	0.011

a Quantitative detection presents the concentrations reported in mg/kg, which are correlated with the concentrations obtained from standardized measurements performed on the same samples by the Finnish Customs.

b The measurement of pineapple extract in positive mode was conducted by injecting 5 μL of the fruit extract.

c The pesticide content of the pineapple fruit swab is reported in relation to the whole pesticide content of the fruit determined by the Finnish Customs using the standardized procedures. This approach was followed as the actual pesticide amount present in the surface of the fruit was unknown.

**Figure 2:**
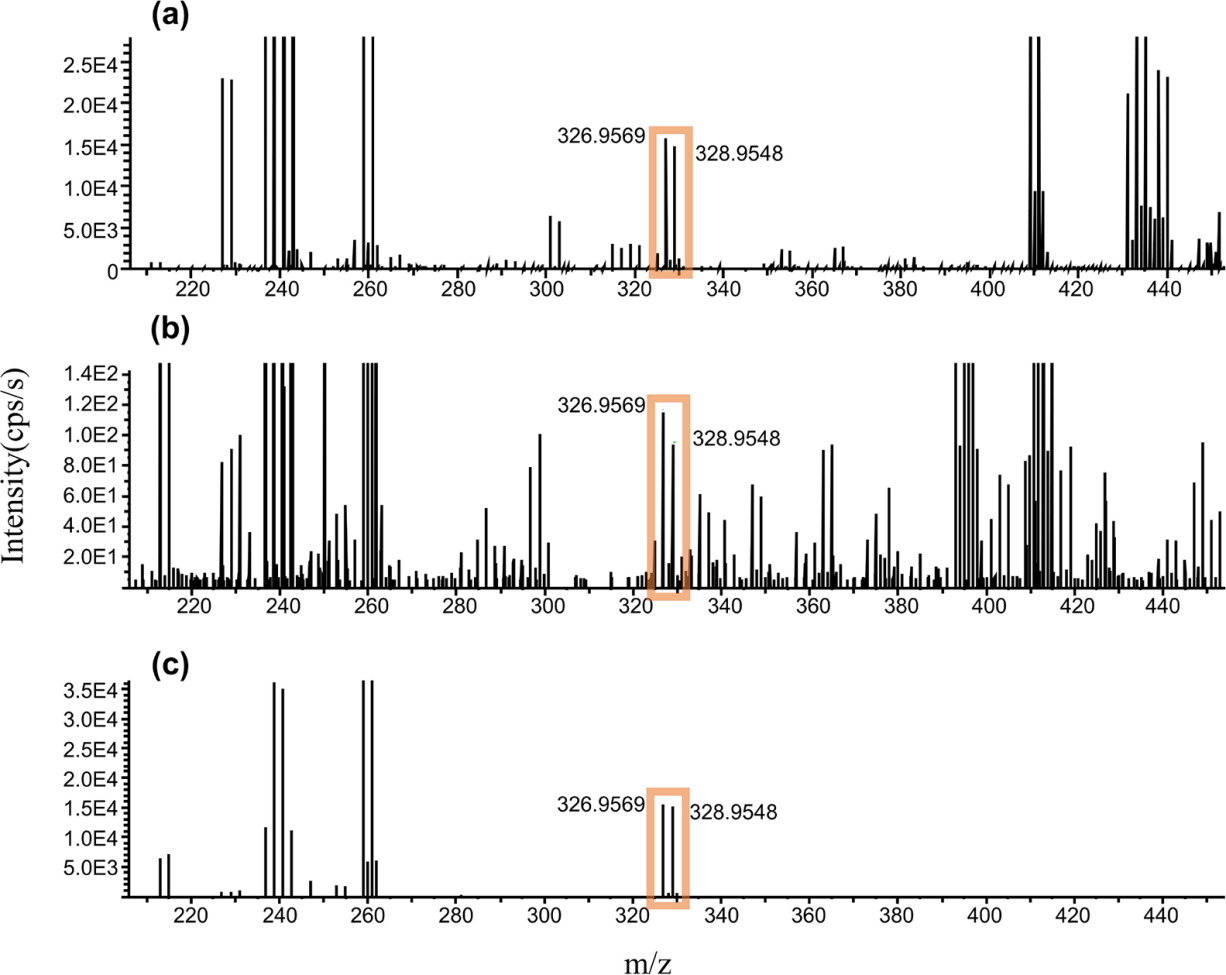
Spectra of fludioxonil adducts with bromide in standard mixture (spectrum a). Fludioxonil adducts with bromide in pineapple juice (spectrum b). Fludioxonil adducts with bromide on the surface of pineapple (spectrum c).

Qualitative identification of the pesticide residues in fruit extracts and pineapple samples (surface of the fruit and juice exuded from slices) was based on the detection of target adducts with bromide in negative polarity and protonated targets in positive polarity (i.e., H_3_O^+^ ionization by radiating the air feed). It resulted in the detection of dimethomorph, dinotefuran, imazalil, azoxystrobin, thiabendazole, fludioxonil, and diazinon, all below their MRL. Table 3 shows the results of these measurements (see Supporting Information Table S4 for more information). Due to different signal intensities of the parent peaks in non-identical samples, different numbers of isotopes appear and match, as explained above (i.e., number of isotopes of fludioxonil in pineapple extract appears different here in comparison to the pineapple swabbing experiment). The fludioxonil bromide adducts detected from standard mixture, pineapple juice and on the surface of the pineapple fruit collected by the swabbing method, are illustrated in Figure 2.

The swabbing method employed in this study offers the advantage of bypassing laborious sample preparation steps typically required for detecting pesticides. With swabbing, the surface of goods or samples can be directly probed, eliminating the need for extensive extraction or cleanup procedures. This approach saves time and resources while still allowing for effective detection of pesticide residues. The sampling process takes only around 30 s, and the recording of the TD profile of the sample requires approximately 4 min. In chromatographic techniques, the usage of different separation conditions for each sample type can be challenging and time-consuming. In traditional analytical methods, a separate column is often required for each sample to prevent cross-contamination and ensure accurate results.^25,26^ This necessitates frequent column changes or running parallel analysis methods, which increases the complexity and cost of the analytical process. In contrast, the swabbing method described in this study utilizes a single system without chromatographic separation. By leveraging the swabbing method with a filter-based approach, the study streamlines the analytical workflow, making it more convenient and efficient for detecting pesticides on various surfaces.

Conceivable matrix effects potentially affecting the ionization performance during swab sampling could result from the sample matrix containing large amounts of strong acids, for example, which could affect the charge transfer to the sampled pesticides. Similarly, very wet samples could provide a challenge for the halogen-based ionization schemes, which are known to be affected by the sample water content.^23^’^27^

**Figure 3:**
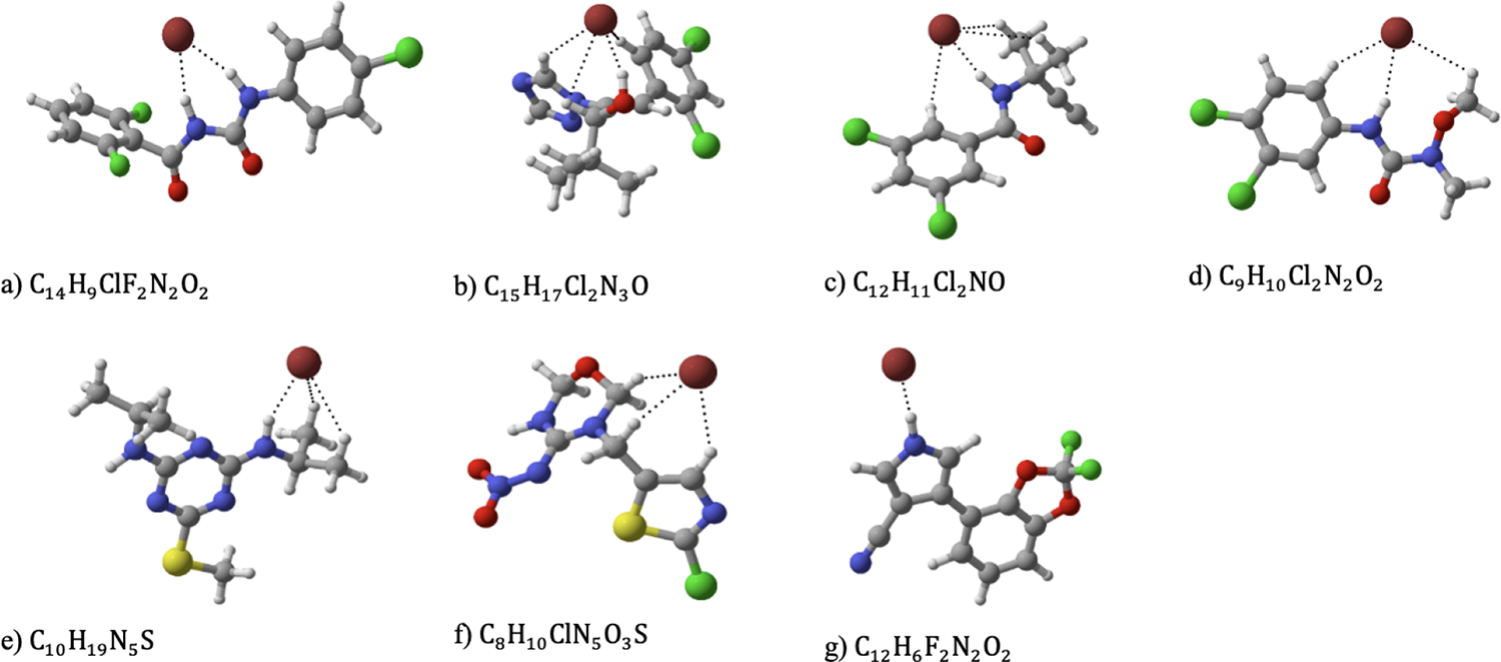
The identified lowest-energy structures of bromide adducting with (a) diflubenzuron, (b) diniconazole, (c) propyzamide, (d) linuron, (e) prometryn, (f) thiamethoxam, and (g) fludioxonil.

**Figure 4:**
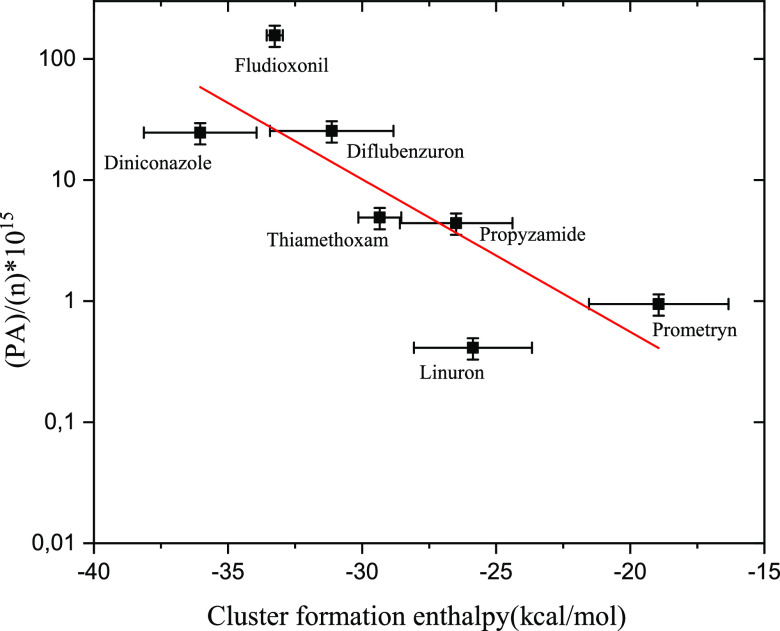
Detection sensitivity as a function of the reagent ion–target molecule binding strength. The pesticide-bromide adduct formation enthalpies plotted against the measured desorption profile PAs normalized by the molar concentrations of the targets. For the numerical values, see Supporting Information Table S5.

Eight pesticides that formed adducts with Br^–^ were selected for a further computational investigation. The selection contained different pesticides in terms of usage and chemical functional groups (see Figure 3 and Supporting information Table S3). The quantum chemically calculated adduct formation enthalpies (i.e., inverse of the adduct binding strength) were plotted against the detection sensitivity (i.e., the integrated thermal desorption profile PA normalized by the molar concentration of the pesticide), which resulted in a near linear correlation between the quantities (Figure 4). A similar correlation of adduct binding strength and detection sensitivity has been observed previously for I– adducts.^28^

Molecular properties such as the size and the polarity of the sample molecule influence the adduct binding strength. In addition, the spatial orientation of the molecule during the initial collision with the Br– ion, together with the shielding of the binding site by bulky substituents, likely affects the fraction of collisions leading to stable adducts—and consequently the instrument sensitivity. Moreover, other factors such as entropy are affecting adduct formation and thus these unaccounted influences are likely to explain the outliers in Figure 4. In general, the quantum chemical calculations support the experimental results, and the detection sensitivity improves as a function of the formation enthalpy (i.e., the adduct binding strength), even at the low level of theory used here, necessitated by the large sizes of the studied systems.

Bromide ions are commonly known to form strong bonds with target molecules by accepting hydrogen bonds. In most cases, bromide ions tend to form strong hydrogen bonds with hydroxyl groups (−OH) present in the target pesticide. However, when hydroxyl groups are absent, bromide ions can form hydrogen bonds with amino groups (−NH) instead (refer to Figure 3).

**Figure 5:**
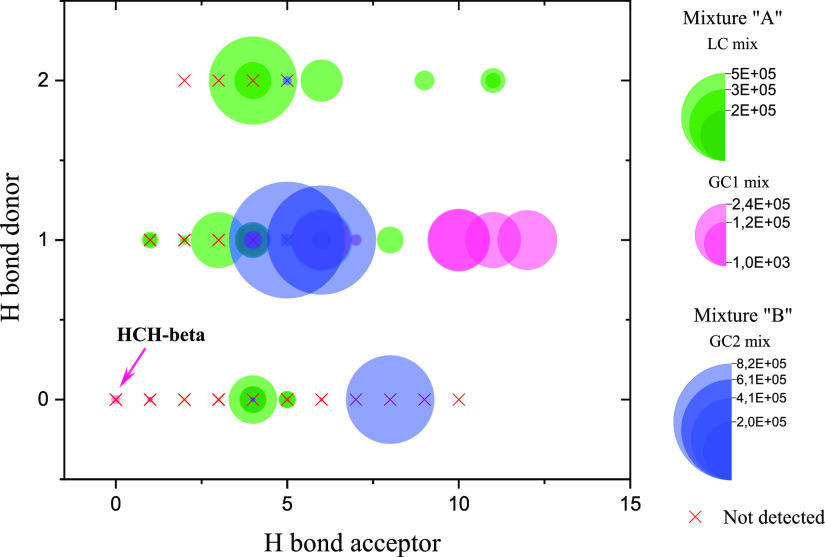
The number of hydrogen bond donating (HBD) sites as a function of hydrogen bond accepting (HBA) sites of all pesticides studied in this work. Red cross indicates the “not detected” compounds. The surface of the circle indicates the desorption profile PA determined for each pesticide. Mixture “A” is a combination of LC and GC1 mixtures.

To be able to observe the correlation between a strong enough HBD site and making an adduct with bromide, for each pesticide molecule, the number of HBD and HBA sites was counted using an automated routine in ChemDraw 20.1.1. Figure 5 demonstrates the number of HBD vs HBA sites by this routine of all the inspected pesticides in different mixtures. Interestingly, the compounds detected in this study tend to have a higher number of HBA sites compared to HBD sites, which is somewhat unexpected. Overall, 86% of the molecules, which made an adduct with bromide, have at least four HBA sites while 75% have at least one HBD site.

**Figure 6:**
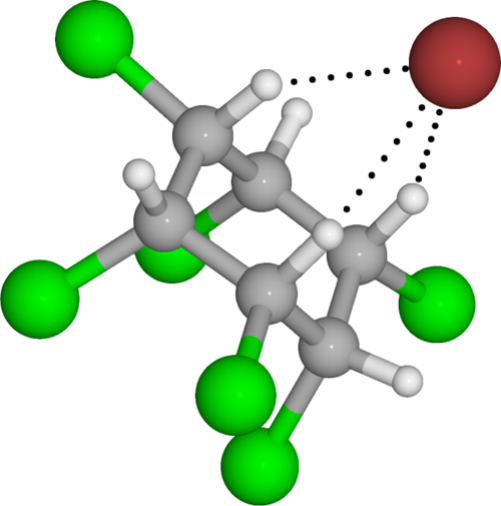
The beta-hexachlorocyclohexane (HCH-beta) bromide adduct configuration. Color coding: carbon—gray, hydrogen—white, chlore—green, bromide—red.

This finding challenges the common understanding of efficient ion binding. For example, in the case of iodide (I−) binding with oxidized compounds, efficient binding can occur with even one hydrogen bond donor (HBD) site.^28^ The apparent correlation between a higher number of HBA sites and bromide binding can be explained by the fact that HBA sites act as electron-withdrawing groups, which can enhance the strength of adjacent HBD sites. This phenomenon likely contributes to the unexpected result observed. Nevertheless, Figure 5 shows that a bromide reagent ion can even bind into species that have no apparent HBD or HBA sites, the binding in these cases likely provided by several similar yet weaker electrostatic interactions (see Figure 6 for an example). Overall, these findings highlight the complex nature of bromide ion binding and suggest that it can occur through various interactions, including both strong hydrogen bonding and several weaker electrostatic interactions.

One of the compounds detected without apparent HBD or HBA sites is the beta-hexachlorocyclohexane (C_6_H_6_Cl_6_) from GC1 solution (i.e., from mixture “A”; shown by arrow in Figure 5). The chair configuration of this molecule, where all the hydrogen atoms are on the same side of the ring, with three of them pointing out of the same plane, leads to a favorable adduct geometry with the bromide ion and subsequent detection as a bromide adduct (Figure 6). The bonding of this apparent outlier is likely explained by numerous similar, yet more weak and subtle hydrogen bonding interactions between the bromide ion and the (Cl−)C–H-bonded hydrogens.

## Conclusions

In this study, a large collection of pesticide standards and a selection of authentic food samples containing pesticide residues were investigated by CIMS applying several concomitant ion schemes. A thermal desorption unit coupled to a multi-scheme chemical ionization source (TD-MION) and an orbitrap mass spectrometer were utilized for the laboratory characterization. The results show a promising method devoid of laborious sample preparation for fast screening of common food samples. The application of controlled chemical ionization with carefully chosen ion schemes can provide a means for quantifying various pesticides with a multitude of functional groups, in a semi-simultaneous routine.

The main goal of this study was to introduce the methodology and to determine the performance of the selected ions toward each pesticide. Rapid pesticide screening from standard solutions as well as from untreated samples (fruit slices and fruit juice) was demonstrated, with a total of 115 pesticides out of 208 detected from the standard solutions containing 1 ng/μL. Additionally, bromide ionization chemistry was further inspected theoretically by determining quantum chemical adduct binding enthalpies for a carefully selected subgroup of targets. This was accomplished to provide important structural insights into Br– adducting at the molecular level and to investigate the previously found correlation between detection sensitivity and the target molecule–reagent ion interaction strength. A similar correlation was found also for the pesticides researched in this work. Interestingly, bromide ion was observed to bind even into weakly hydrogen bonding sites, somewhat contrary to the expectations from previous studies.

## Supporting Information Available

Thermal desorption profile of fludioxonil from pineapple pesticide extract, complete lists of pesticides in mixtures “A” and “B” with their respective detection details, information on the pesticides chosen for the computational study, integrated thermal desorption profile peak area (PA) and detection parameters of detected pesticides, adduct formation enthalpies and normalized peak areas, corrections to adduct formation enthalpies, and isotope spectra of various pesticides in different fruit extracts (PDF)
