# Supplementary material for: Pesticide Residue Fast Screening Using Thermal Desorption Multi-Scheme Chemical Ionization Mass Spectrometry (TD-MION MS) with Selective Chemical Ionization
Source: ACS Omega. 2023 Jul 15;8(29):25749–57. doi: 10.1021/acsomega.3c00385 (PMC10373215; doi:10.1021/acsomega.3c00385)
Supplement: Supplementary file 1 — ao3c00385_si_001.pdf [file ao3c00385_si_001.pdf]

Pesticide Residue Fast Screening Using Thermal Desorption Multi-Scheme Chemical Ionization Mass Spectrometry (TD MION MS) with Selective Chemical Ionization

Fariba Partovi[1,2], Joona Mikkilä[1,*], Siddharth Iyer[2], Jyri Mikkilä[1], Jussi Kontro[1], Suvi Ojanperä[3], Paxton Juuti[1], Juha Kangasluoma[1,4], Aleksei Shcherbinin[1], Matti Rissanen[2,5,*]

[1]Karsa Ltd., A. I. Virtasen aukio 1, 00560 Helsinki, Finland

[2]Aerosol Physics Laboratory, Physics Unit, Faculty of Engineering and Natural Sciences, Tampere University, 33720 Tampere, Finland

[3]Finnish Customs, P.O. Box 512, FI-00101 Helsinki, Finland

[4]Institute for Atmospheric and Earth System Research/Physics, Faculty of Science, University of Helsinki, 00014 Helsinki, Finland

[5]Department of Chemistry, University of Helsinki, 00014 Helsinki, Finland

Supporting Information

Table of Contents

Figure S1. Thermal desorption profile of Fludioxonil from pineapple pesticide extract…………………**S3**

Table S1. Complete list of pesticides in Mixture "A" and their detection details (Target name, chemical formula, reagent used, adduct type, peak area)…………………………………………………………**S3**

Table S2. Complete list of pesticides in Mixture "B" and their detection details (Target name, chemical formula, reagent used, adduct type, peak area)…………………………………………………………**S7**

Table S3. Detailed information of the pesticides selected for the computational study (Name, chemical structure and formula, usage, number of H bond donor and H bond acceptor…………………………….**S9**

Table S4. The integrated thermal desorption profile peak area (PA) of the detected pesticides as well as various detection parameters (Adduct type, number of isotopes matched, isotopic pattern score (%), Quantitative detection(mg/kg))……………………………………………………………………...**S10**

Table S5. Adduct formation enthalpies and the integrated thermal desorption profile peak areas (PA) normalized by molar concentration of the detected bromide adducts (n)……………………………..**S10**

Table S6. DLPNO-CCSD(T)/aug-cc-pVTZ-PP corrections to the B3LYP/6-31+G(d) calculated adduct formation enthalpies. Negative corrections indicate lower formation enthalpies than those predicted by the B3LYP/6-31+G(d) method…………………………………………………………………….**S11**

Figure S2. Isotope spectra of protonated dimethomorph in watermelon extract. Expected isotope peaks in blue, detected isotope peaks in red. (a) overall isotope spectra, (b) zoomed-in view of individual peaks…………………………………………………………………………………………...**S12**

Figure S3. Isotope spectra of protonated imazalil in watermelon extract. Expected isotope peaks in blue, detected isotope peaks in red. (a) overall isotope spectra, (b) zoomed-in view of individual peaks…….**S13**

Figure S4. Isotope spectra of dinotefuron bromide adduct in watermelon extract. Expected isotope peaks in blue, detected isotope peaks in red. (a) overall isotope spectra, (b) zoomed-in view of individual peaks…………………………………………………………………………………………...**S14**

Figure S5. Isotope spectra of protonated azoxystrobin in lime extract. Expected isotope peaks in blue, detected isotope peaks in red. (a) overall isotope spectra, (b) zoomed-in view of individual peaks…….**S15**

Figure S6. Isotope spectra of protonated imazalile in lime extract. Expected isotope peaks in blue, detected isotope peaks in red. (a) overall isotope spectra, (b) zoomed-in view of individual peaks………………...**S16**

Figure S7. Isotope spectra of protonated thiabendazole in avocado extract. Expected isotope peaks in blue, detected isotope peaks in red. (a) overall isotope spectra, (b) zoomed-in view of individual peaks…………………………………………………………………………………………...**S17**

Figure S8. Isotope spectra of protonated diazinone in pineapple extract. Expected isotope peaks in blue, detected isotope peaks in red. (a) overall isotope spectra, (b) zoomed-in view of individual peaks…….**S18**

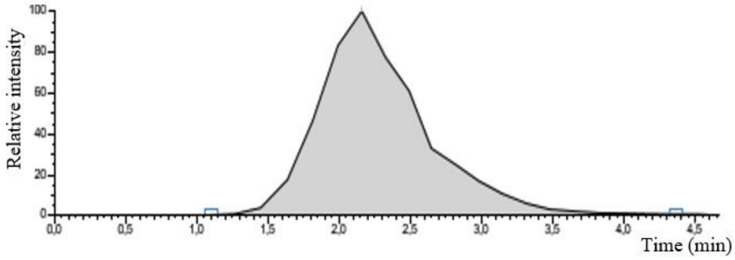

Figure S1 Thermal desorption profile of Fludioxonil from pineapple pesticide extract. The thermal desorption is a heat exchange process to vaporize or volatize components, in this study, from the surface of the filter containing the sample.

Table S1 Complete list of pesticides in Mixture "A" and their detection details.

Mixture "A"

GC1 mix								
Reagent		DBrME		Acac		Air		
		Br-	H loss	Acac[a]+H	H adduct	H adduct	H loss	
Target Name	Formula	Peak Area[b]						
2-Phenyl phenol	C12H10O	2.72E+02	N/A[c]	N/A	N/A	N/A	1.26E+02	
Aclonifen	C12H9ClN2O3	N/A	N/A	N/A	N/A	N/A	N/A	
Acrinathrin	C26H21F6NO5	1.85E+03	N/A	N/A	N/A	N/A	1.01E+03	
Anthraquinone	C14H8O2	N/A	N/A	N/A	1.01E+03	1.16E+02	N/A	
Atrazine	C8H14ClN5	N/A	N/A	N/A	1.17E+05	3.67E+04	N/A	
Bifenthrin	C23H22ClF3O2	N/A	9.54E+02	N/A	N/A	N/A	N/A	
Biphenyl	C12H10	N/A	N/A	N/A	N/A	N/A	N/A	
Bromofos-ethyl	C10H12BrCl2O3PS	N/A	N/A	N/A	N/A	N/A	N/A	
Bromofos-methyl	C8H8BrCl2O3PS	N/A	N/A	N/A	N/A	N/A	N/A	
Captan	C9H8Cl3NO2S	N/A	1.83E+02	N/A	N/A	N/A	N/A	
Chlorfenapyr	C15H11BrClF3N2O	1.01E+03	N/A	N/A	N/A	N/A	N/A	
Chlormephos	C5H12ClO2PS2	N/A	N/A	N/A	N/A	N/A	N/A	
Chlorpyrifos	C9H11Cl3NO3PS	N/A	N/A	N/A	1.98E+03	6.99E+02	N/A	
chlorthal-dimethyl	C10H6Cl4O4	N/A	N/A	N/A	N/A	N/A	N/A	
chlozolinate	C13H11Cl2NO5	N/A	N/A	N/A	N/A	8.49E+01	N/A	
Chlropyrifos-methyl	C7H7Cl3NO3PS	N/A	N/A	N/A	2.21E+02	3.46E+02	N/A	
Cypermethrin	C22H19Cl2NO3	N/A	N/A	N/A	N/A	N/A	N/A	
DDD-p,p	C14H10Cl4	N/A	N/A	N/A	N/A	N/A	N/A	
DDE-p,p	C14H8Cl4	N/A	N/A	N/A	N/A	N/A	N/A	
DDT-o,p	C14H9Cl5	N/A	N/A	N/A	N/A	N/A	N/A	
DDT-p,p	C14H9Cl5	N/A	N/A	N/A	N/A	N/A	N/A	

[a] *Acac: Acetonylacetone*

[b] *Peak area refers to the area under the thermal desorption profile of a compound and is given in an arbitrary unit. The red colour compounds are not detected at all. The green colour indicates "confirmed detection" at least with one mode of detection. The yellow colour indicates "detected" compounds with at least one mode of detection.*

[c] *N/A: Not applicable (Here used for "not detected" peak area)*

Deltamethrin	C22H19Br2NO3	N/A	N/A	N/A	N/A	N/A	N/A
Diazinon	C12H21N2O3PS	1.70E+03	N/A	N/A	1.43E+05	2.24E+04	N/A
Dichlofluanid	C9H11Cl2FN2O2S2	N/A	1.15E+02	N/A	7.15E+02	1.70E+02	N/A
Dicloran	C6H4Cl2N2O2	N/A	N/A	N/A	N/A	N/A	N/A
Dicofol-o,p	C14H9Cl5O	N/A	N/A	N/A	N/A	N/A	N/A
Dicofol-p,p	C14H9Cl5O	N/A	N/A	N/A	N/A	N/A	N/A
Dieldrin	C12H8Cl6O	N/A	N/A	N/A	N/A	N/A	N/A
Diphenylamine	C12H11N	N/A	N/A	N/A	3.67E+03	5.64E+03	N/A
Endosulfan-alpha	C9H6Cl6O3S	1.66E+03	N/A	N/A	N/A	N/A	N/A
Endosulfan-beta	C9H6Cl6O3S	1.66E+03	N/A	N/A	N/A	N/A	N/A
Endosulfanesulfate	C9H6Cl6O4S	3.42E+03	N/A	N/A	N/A	N/A	5.20E+03
Fiproniili-sulfide	C12H4Cl2F6N4S	2.37E+05	1.50E+03	N/A	N/A	N/A	1.41E+05
Fiproniili-sulfone	C12H4Cl2F6N4O2S	2.15E+05	6.04E+04	N/A	N/A	N/A	1.76E+05
Fipronil	C12H4Cl2F6N4OS	1.88E+05	1.43E+04	N/A	N/A	N/A	1.24E+05
Fipronil-desulfinyl	C12H4Cl2F6N4	2.26E+05	N/A	N/A	N/A	N/A	1.30E+05
Fluvalinate-tau	C26H22ClF3N2O3	7.38E+03	N/A	N/A	9.33E+02	2.29E+02	5.27E+02
HCH-beta	C6H6Cl6	5.05E+03	N/A	N/A	N/A	N/A	N/A
Heptachlor	C10H5Cl7	N/A	N/A	N/A	N/A	N/A	N/A
Hexachlrobenzene	C6Cl6	N/A	N/A	N/A	N/A	N/A	N/A
Hexaconazole	C14H17Cl2N3O	1.90E+04	N/A	N/A	8.46E+03	3.67E+03	N/A
Isofenphos	C15H24NO4PS	N/A	N/A	N/A	4.17E+03	1.62E+03	1.79E+02
Isofenphos-methyl	C14H22NO4PS	N/A	N/A	N/A	2.45E+03	1.46E+03	N/A
Isoprothiolane	C12H18O4S2	N/A	N/A	1.37E+02	6.77E+04	1.38E+04	N/A
Kresoxim-methyl	C18H19NO4	N/A	N/A	N/A	4.81E+03	2.54E+03	N/A
Metrafenone	C19H21BrO5	N/A	N/A	N/A	1.03E+04	3.90E+03	N/A
Nitrothal-isopropyl	C14H17NO6	N/A	N/A	N/A	N/A	N/A	N/A
Pendimethalin	C13H19N3O4	N/A	N/A	N/A	1.42E+03	1.45E+03	N/A
Pentachloroaniline	C6H2Cl5N	N/A	N/A	N/A	N/A	N/A	N/A
Pentachloroanisole	C7H3Cl5O	N/A	N/A	N/A	N/A	N/A	N/A
Penthiopyrad	C16H20F3N3OS	2.07E+05	9.29E+01	N/A	1.27E+04	4.64E+03	4.84E+04
Pirimiphos-methy	C11H20N3O3PS	N/A	N/A	N/A	1.68E+05	3.46E+04	N/A
Profenofos	C11H15BrClO3PS	N/A	N/A	N/A	1.39E+03	5.51E+02	N/A
Propoxur	C11H15NO3	N/A	N/A	N/A	1.16E+04	4.53E+03	N/A
Pyridaben	C19H25ClN2OS	N/A	N/A	4.66E+02	1.37E+04	4.05E+03	N/A
Quinalphos	C12H15N2O3PS	N/A	N/A	2.15E+03	3.88E+04	8.84E+03	N/A
Quintozene	C6Cl5NO2	N/A	N/A	N/A	N/A	N/A	N/A
Sulfotep	C8H20O5P2S2	N/A	3.17E+02	N/A	1.89E+04	4.47E+03	N/A
Terbuthylazine	C9H16ClN5	N/A	N/A	2.39E+02	9.80E+04	2.62E+04	N/A
Tetradifon	C12H6Cl4O2S	N/A	N/A	N/A	N/A	N/A	N/A
Tetrahydrophthalimide	C8H9NO2	N/A	N/A	N/A	N/A	3.51E+02	1.17E+02
Tetramethrin	C19H25NO4	N/A	N/A	N/A	5.49E+03	2.43E+03	N/A
Tolfenpyrad	C21H22ClN3O2	N/A	N/A	N/A	8.06E+03	2.48E+03	1.57E+02
Trichloronat	C10H12Cl3O2PS	N/A	N/A	N/A	N/A	N/A	N/A
Vinclozolin	C12H9Cl2NO3	N/A	N/A	N/A	N/A	N/A	N/A

LC mix

Acetamiprid	C10H11ClN4	4.36E+04	N/A	N/A	7.43E+04	1.98E+04	1.86E+03
Aldicarb	C7H14N2O2S	N/A	N/A	N/A	N/A	N/A	N/A
Amitraz	C19H23N3	N/A	N/A	N/A	1.03E+05	3.42E+04	N/A
Boscalid	C18H12Cl2N2O	5.44E+03	N/A	N/A	3.93E+03	1.85E+03	4.51E+04
Bupirimate	C13H24N4O3S	N/A	N/A	N/A	2.42E+05	8.01E+04	N/A
Buprofezin	C16H23N3OS	N/A	N/A	3.32E+02	2.57E+05	6.65E+04	N/A
Carbaryl	C12H11NO2	N/A	N/A	1.85E+02	5.39E+03	2.07E+03	N/A
Carbenazim	C9H9N3O2	N/A	N/A	3.94E+02	1.47E+05	3.59E+04	3.22E+03
Carbofuran	C12H15NO3	N/A	N/A	1.91E+02	5.37E+04	1.59E+04	N/A
Chlorantraniliprole	C18H14BrCl2N5O2	7.72E+03	2.22E+03	N/A	4.32E+03	1.30E+03	3.55E+03
Difenoconazole	C19H17Cl2N3O3	N/A	N/A	N/A	2.15E+04	6.03E+03	N/A
Diflubenzuron	C14H9ClF2N2O2	8.19E+04	3.11E+02	N/A	2.90E+03	1.31E+03	1.33E+05
Dimethoate	C5H12NO3PS2	1.11E+04	N/A	1.26E+04	3.97E+04	1.15E+04	N/A
Dimethomorph	C21H22ClNO4	3.94E+02	N/A	N/A	8.00E+04	1.25E+04	N/A
Diniconazole	C15H17Cl2N3O	7.54E+04	N/A	N/A	1.30E+04	3.72E+03	N/A
Dinotefuran	C7H14N4O3	4.65E+05	2.89E+02	5.75E+02	9.16E+04	3.58E+04	2.71E+05
Disulfoton	C8H19O2PS3	N/A	N/A	2.33E+03	N/A	1.75E+02	N/A
Ethiofencarb	C11H15NO2S	N/A	N/A	N/A	2.71E+04	8.37E+03	N/A
Famoxadone	C22H18N2O4	N/A	8.38E+03	N/A	N/A	N/A	1.35E+03
Fenamiphos	C13H22NO3PS	2.40E+02	N/A	N/A	4.85E+04	1.41E+04	N/A
Fenarimol	C17H12Cl2N2O	N/A	N/A	N/A	2.60E+04	7.98E+03	8.42E+02
Fenheksamidi	C14H17Cl2NO2	N/A	N/A	N/A	3.97E+03	7.40E+02	1.93E+04
Fenpyrazamine	C17H21N3O2S	N/A	N/A	N/A	1.82E+05	5.12E+04	4.00E+02
Fensulfothion	C11H17O4PS2	N/A	N/A	N/A	4.32E+04	9.22E+03	N/A
Fenthion	C10H15O3PS2	N/A	N/A	N/A	3.38E+03	1.83E+03	N/A
Flonicamid	C9H6F3N3O	2.31E+05	4.45E+02	N/A	4.55E+03	2.55E+03	2.20E+05
Flubendiamidi	C23H22F7IN2O4S	1.39E+04	N/A	N/A	N/A	N/A	1.98E+04
Flufenuxuron	C21H11ClF6N2O3	2.37E+04	N/A	N/A	3.33E+03	8.45E+02	1.82E+04
Fluopicolide	C14H8Cl3F3N2O	1.22E+03	N/A	N/A	8.72E+03	2.34E+03	2.90E+02
Fluopyram	C16H11ClF6N2O	4.31E+04	N/A	N/A	6.68E+04	1.10E+04	3.14E+03
Flusilazole	C16H15F2N3Si	2.43E+03	N/A	N/A	6.30E+04	1.69E+04	N/A
Flutriafol	C16H13F2N3O	1.97E+04	N/A	N/A	4.68E+04	1.28E+04	2.63E+03
Hexythiazox	C17H21ClN2O2S	N/A	N/A	N/A	2.54E+03	8.96E+02	N/A
Imazalil	C14H14Cl2N2O	N/A	N/A	N/A	1.57E+05	3.87E+04	N/A
Imidacloprid	C9H10ClN5O2	2.72E+04	N/A	N/A	2.25E+04	6.13E+03	8.60E+04
Indoxarcarb	C22H17ClF3N3O7	N/A	N/A	N/A	9.38E+03	3.73E+03	N/A
Linuron	C9H10Cl2N2O2	1.65E+03	N/A	N/A	8.97E+03	1.96E+03	2.91E+03
Lufenuron	C17H8Cl2F8N2O3	3.73E+04	3.80E+03	N/A	1.16E+02	N/A	7.63E+04
Malathion	C10H19O6PS2	N/A	N/A	N/A	1.39E+04	3.41E+03	9.94E+01
Mandipropamid	C23H22ClNO4	7.75E+04	N/A	N/A	1.24E+04	5.99E+03	5.69E+02
Mepanipyrim	C14H13N3	N/A	N/A	2.70E+03	3.57E+05	1.02E+05	N/A
Metalaxyl	C15H21NO4	N/A	N/A	N/A	1.17E+05	2.92E+04	N/A
Methamidophos	C2H8NO2PS	N/A	N/A	N/A	2.09E+04	4.90E+03	1.73E+03

Methiocarb	C11H15NO2S	N/A	N/A	N/A	2.71E+04	8.37E+03	N/A
Methomyl	C5H10N2O2S	N/A	N/A	N/A	1.92E+04	6.58E+03	N/A
Methoxyfenozide	C22H28N2O3	1.87E+05	N/A	N/A	2.02E+04	4.63E+03	9.99E+04
Monocrotophos	C7H14NO5P	1.01E+03	N/A	N/A	2.67E+04	2.23E+04	N/A
Myclobutanil	C15H17ClN4	4.21E+04	N/A	1.01E+02	1.94E+04	5.01E+03	N/A
Oxdixyl	C14H18N2O4	N/A	N/A	N/A	3.12E+04	1.03E+04	N/A
Oxydemeton-methyl	C6H15O4PS2	N/A	N/A	N/A	1.22E+05	3.77E+04	N/A
Penflufen	C18H24FN3O	N/A	N/A	N/A	9.50E+04	3.02E+04	N/A
Phorate	C7H17O2PS3	N/A	N/A	N/A	N/A	1.27E+03	N/A
Phosmet	C11H12NO4PS2	N/A	N/A	N/A	8.14E+03	2.51E+03	N/A
Pirimicarb	C11H18N4O2	N/A	N/A	1.23E+02	3.02E+05	9.49E+04	N/A
Propamocarb	C9H20N2O2	N/A	N/A	2.54E+02	3.63E+05	1.26E+05	N/A
Propargiitti	C19H26O4S	5.62E+02	N/A	N/A	N/A	N/A	N/A
Propiconazole	C15H17Cl2N3O2	N/A	N/A	1.39E+02	2.86E+04	5.85E+03	N/A
Propyzamide	C12H11Cl2NO	1.72E+04	N/A	N/A	2.99E+03	1.66E+03	2.12E+04
Pyraclostrobin	C19H18ClN3O4	N/A	N/A	N/A	1.31E+05	2.07E+04	N/A
Pyrimethanil	C12H13N3	1.30E+03	N/A	N/A	2.94E+05	8.86E+04	N/A
Pyriproxyfen	C20H19NO3	N/A	N/A	N/A	1.58E+05	3.04E+04	N/A
Quinoxyfen	C15H8Cl2FNO	N/A	N/A	1.06E+02	5.25E+04	1.31E+04	N/A
Spinosad (spinosyl A)	C41H65NO10	N/A	N/A	N/A	1.11E+03	1.92E+02	N/A
Spinosad D	C42H67NO10	N/A	N/A	N/A	N/A	N/A	N/A
Spirotetramat	C21H27NO5	N/A	N/A	N/A	9.06E+04	2.19E+04	N/A
Tebuconazol	C16H22ClN3O	6.02E+04	N/A	N/A	6.31E+04	1.63E+04	N/A
Teflubenzuron	C14H6Cl2F4N2O2	1.07E+05	7.44E+03	N/A	N/A	N/A	5.34E+04
Terbufos	C9H21O2PS3	1.17E+02	N/A	N/A	3.06E+02	4.40E+02	N/A
Thiabendazole	C10H7N3S	N/A	N/A	2.38E+02	2.74E+05	8.97E+04	2.42E+03
Thiacloprid	C10H9ClN4S	1.40E+05	N/A	N/A	4.65E+04	8.92E+03	N/A
Thiamethoxam	C8H10ClN5O3S	1.68E+04	N/A	N/A	2.31E+04	6.20E+03	N/A
Triadimefon	C14H16ClN3O2	1.81E+04	N/A	N/A	3.07E+04	7.14E+03	1.03E+04
Trifloxystrobin	C20H19F3N2O4	N/A	2.83E+03	6.73E+02	7.78E+04	1.84E+04	1.01E+02

Table S2 Complete list of pesticides in Mixture "B" and their detection details.

Mixture "B"								
GC2								
Reagent		DBrME		Acac		H2O		
		Br-	H loss	Acac[a]+H	H adduct	H adduct	H loss	
Target Name	Formula	Peak Area[b]						
Aldrin	C12H8Cl6	N/A[c]	N/A	N/A	N/A	N/A	N/A	
alpha-Cypermethrin	C22H19Cl2NO3	N/A	N/A	N/A	N/A	N/A	N/A	
alpha-HCH	C6H6Cl6	N/A	N/A	N/A	N/A	N/A	N/A	
Azoxystrobin	C22H17N3O5	N/A	N/A	N/A	7.90E+03	N/A	N/A	
Bifenazate	C17H20N2O3	N/A	N/A	N/A	N/A	N/A	N/A	
Bifenazate-diazene	C17H18N2O3	N/A	N/A	N/A	6.16E+03	6.94E+02	N/A	
Bitertanol	C20H23N3O2	9.43E+03	N/A	N/A	1.60E+03	N/A	3.22E+02	
Bromopropylate	C17H16Br2O3	N/A	N/A	N/A	N/A	N/A	N/A	
Chlorbufam	C11H10ClNO2	N/A	N/A	N/A	N/A	N/A	N/A	
Chlorfenvinphos	C12H14Cl3O4P	N/A	N/A	N/A	4.38E+02	3.23E+02	N/A	
Chlorpropham	C10H12ClNO2	N/A	N/A	N/A	N/A	N/A	N/A	
Chlorpyrifos-methyl	C7H7Cl3NO3PS	N/A	N/A	N/A	1.91E+02	4.59E+02	N/A	
Cinerin II	C21H28O5	1.68E+04	N/A	N/A	N/A	N/A	N/A	
Cinerin I	C20H28O3	1.01E+03	N/A	N/A	1.40E+02	1.08E+02	1.56E+03	
cis-Heptachlor Epoxide	C10H5Cl7O	N/A	N/A	N/A	N/A	N/A	N/A	
Cyflufenamid	C20H17F5N2O2	3.39E+02	3.51E+02	N/A	1.91E+03	1.76E+03	3.99E+02	
Cyfluthrin	C22H18Cl2FNO3	1.18E+03	N/A	N/A	N/A	N/A	7.57E+01	
Cyprodinil	C14H15N3	1.06E+02	N/A	N/A	6.32E+04	5.88E+03	N/A	
Dicholobenil	C7H3Cl2N	N/A	N/A	N/A	N/A	N/A	N/A	
Endrin	C12H8Cl6O	N/A	N/A	N/A	N/A	N/A	N/A	
Esfenvalerate	C25H22ClNO3	N/A	1.99E+02	N/A	N/A	N/A	N/A	
Ethion	C9H22O4P2S4	2.79E+02	N/A	N/A	9.87E+02	1.97E+03	N/A	
Ethoprophos	C8H19O2PS2	N/A	N/A	N/A	6.68E+03	3.95E+03	N/A	
Etofenprox	C25H28O3	6.33E+02	N/A	N/A	N/A	N/A	N/A	
Etrimfos	C10H17N2O4PS	N/A	6.11E+03	N/A	6.75E+04	7.23E+03	N/A	
Fenazaquin	C20H22N2O	2.54E+02	N/A	N/A	2.36E+04	1.51E+03	N/A	
Fenchlorphos	C8H8Cl3O3PS	N/A	N/A	N/A	N/A	N/A	N/A	
Fenitrothion	C9H12NO5PS	N/A	N/A	N/A	N/A	N/A	N/A	
Fenpropathrin	C22H23NO3	N/A	N/A	N/A	N/A	N/A	N/A	
Fludioxonil	C12H6F2N2O2	7.14E+05	1.07E+05	N/A	N/A	N/A	2.50E+05	
Folpet	C9H4Cl3NO2S	N/A	N/A	N/A	N/A	N/A	N/A	
Iprodione	C13H13Cl2N3O3	N/A	9.02E+01	N/A	N/A	N/A	N/A	

[a] *Acac: Acetonylacetone*

[b]*Peak area refers to the area under the thermal desorption profile of a compound and is given in an arbitrary unit. The red colour compounds are not detected at all. The green colour indicates "confirmed detection" at least with one mode of detection. The yellow colour indicates "detected" compounds with at least one mode of detection.*

[c] *N/A: Not applicable (Here used for "not detected" peak area*

Isocarbophos	C11H16NO4PS	N/A	8.77E+01	N/A	N/A	N/A	N/A
Isopyrazam	C20H23F2N3O	8.18E+05	N/A	N/A	3.42E+03	N/A	3.81E+04
Jasmolin II	C22H30O5	1.34E+04	N/A	N/A	N/A	N/A	N/A
Jasmolin I	C21H30O3	3.39E+04	N/A	N/A	N/A	N/A	N/A
lambda-Cyhalothrin	C23H19ClF3NO3	3.98E+02	N/A	N/A	N/A	N/A	N/A
Lindane	C6H6Cl6	N/A	N/A	N/A	N/A	N/A	N/A
Mecarbam	C10H20NO5PS2	N/A	1.52E+02	N/A	3.04E+03	1.16E+03	N/A
Methacrifos	C7H13O5PS	N/A	N/A	N/A	3.33E+02	2.22E+03	N/A
Metolachlor	C15H22ClNO2	N/A	N/A	N/A	2.32E+04	N/A	2.77E+03
Metribuzin	C8H14N4OS	N/A	N/A	N/A	1.93E+04	2.08E+03	N/A
Mevinphos	C7H13O6P	N/A	N/A	N/A	2.45E+03	1.69E+03	N/A
DMST	C9H14N2O2S	1.19E+02	N/A	N/A	6.37E+02	7.98E+02	2.20E+03
Nitrofen	C12H7Cl2NO3	N/A	1.60E+02	N/A	N/A	N/A	N/A
Parathion-ethyl	C10H14NO5PS	N/A	N/A	N/A	N/A	N/A	N/A
Parathion-methyl	C8H10NO5PS	N/A	5.12E+02	N/A	N/A	N/A	N/A
Penconazole	C13H15Cl2N3	4.44E+02	N/A	N/A	1.84E+03	5.77E+02	N/A
Permethrin	C21Cl2H20O3	N/A	N/A	N/A	N/A	N/A	N/A
Phenthoate	C12H17O4PS2	N/A	1.18E+02	N/A	9.14E+02	1.98E+03	N/A
Phthalimide	C8H5NO2	N/A	1.22E+02	N/A	1.00E+02	N/A	7.38E+02
Pirimiphos-ethyl	C13H24N3O3PS	N/A	N/A	N/A	6.79E+04	2.98E+03	N/A
Procymidone	C13H11Cl2NO2	N/A	N/A	N/A	N/A	N/A	N/A
Prometryn	C10H19N5S	4.65E+03	N/A	N/A	7.24E+04	4.50E+03	3.65E+02
Propazine	C9H16N5Cl	7.76E+01	N/A	N/A	9.12E+04	5.16E+03	N/A
Propham	C10H13NO2	N/A	N/A	N/A	N/A	N/A	N/A
Proquinazid	C14H17IN2O2	N/A	N/A	N/A	3.07E+03	2.98E+03	N/A
Prothiofos	C11H15Cl2O2PS2	N/A	N/A	N/A	N/A	N/A	N/A
Pyrazophos	C14H20N3O5PS	N/A	N/A	N/A	2.33E+03	7.77E+02	N/A
Pyrethrin II	C22H28O5	3.59E+04	N/A	N/A	N/A	N/A	N/A
Pyrethrin I	C21H28O3	5.63E+04	N/A	N/A	4.08E+02	7.27E+02	N/A
Sebuthylazine	C9H16ClN5	7.76E+01	N/A	N/A	9.12E+04	5.16E+03	N/A
Tecnazene	C6HCl4NO2	N/A	N/A	N/A	N/A	N/A	N/A
Tefluthrin	C17H14ClF7O2	N/A	1.26E+02	N/A	N/A	N/A	N/A
Tetraconazole	C13H11Cl2F4N3O	4.71E+05	N/A	N/A	8.31E+02	1.46E+02	N/A
Tolclofos-methyl	C9H11Cl2O3PS	N/A	N/A	N/A	1.48E+02	N/A	N/A
Tolylfluanid	C10H13Cl2FN2O2S2	N/A	N/A	N/A	1.07E+02	1.71E+02	N/A
trans-Heptachlor Epoxide	C10H5Cl7O	N/A	N/A	N/A	N/A	N/A	N/A
Triazophos	C12H16N3O3PS	N/A	N/A	N/A	1.77E+04	2.26E+03	N/A
Trifluralin	C13H16F3N3O4	N/A	N/A	N/A	2.56E+02	6.95E+02	N/A

Table S3 Detailed information of the pesticides selected for the computational study.

Name	Chemical structure	Chemical formula	Usage	HBD	HBA
Diniconazole	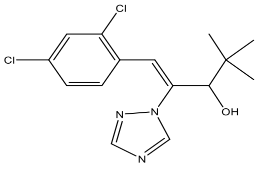	$C_{15}H_{17}Cl_2N_3O$	Fungicide	1	4
Diflubenzuron	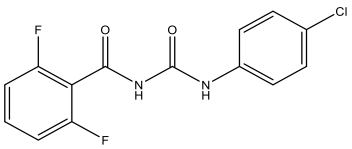	$C_{14}H_9ClF_2N_2O_2$	Insecticide	2	4
Fludioxonil	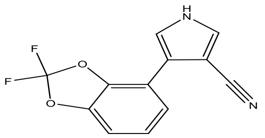	$C_{12}H_6F_2N_2O_2$	Fungicide	1	6
Linuron	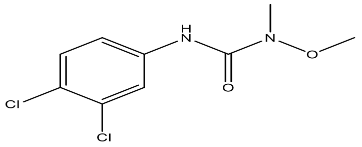	$C_9H_{10}Cl_2N_2O_2$	Herbicide	1	2
Prometryn	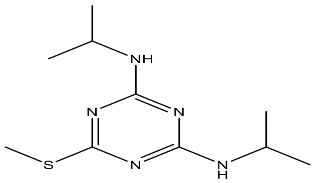	$C_{10}H_{19}N_5S$	Herbicide	2	5
Propyzamide	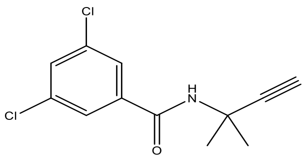	$C_{12}H_{11}Cl_2NO$	Herbicide	1	1
Thiamethoxam	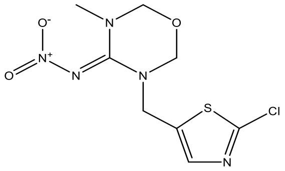	$C_8H_{10}ClN_5O_3S$	Insecticide	0	5

Table S4 The integrated thermal desorption profile peak area (PA) of the detected pesticides as well as various detection parameters

	Adduct	Peak area	Number of isotopes matched	Isotopic pattern score (%)	Quantitative detection (mg/kg)
Watermelon extract					
Dimethomorph	+H+	8.79E2	3 of 3	75	0.010
Dinotefuran	*Br-	2.41E3	4 of 4	100	0.011
Imazalil	+H+	1.12E4	6 of 7	77	0.83
Lime extract					
Azoxystrobin	+H+	1.74E3	2 of 2	87	0.022
Imazalil	+H+	1.09E5	7 of 11	92	1.5
Avocado extract					
Thiabendazole	+H+	1.57E5	5 of 9	91	0.51
Pineapple extract					
Fludioxonil	*Br-	5.35E5	6 of 8	96	0.48
Diazinon	+H+	1.1E3	1 of 2	37	0.011
Pineapple swabbing					
Fludioxonil	*Br-	4.2E5	8 of 11	99	0.48
Diazinon	+H+	7.9E2	2 of 2	100	0.011
Pineapple juice					
Fludioxonil	*Br-	4.4E3	4 of 4	85	0.48
Diazinon	+H+	1.1E2	1 of 1	84	0.011

Table S5 Adduct formation enthalpies and the integrated thermal desorption profile peak areas (PA) normalized by molar concentration of the detected bromide adducts (n)

Name	Formula	Adduct formation enthalpy (Kcal/mol)	(PA)/(n)
Diniconazole	$C_{15}H_{17}Cl_2N_3O$	-36.03596	2.45955E+16
Diflubenzuron	$C_{14}H_9ClF_2N_2O_2$	-31.13260	2.54447E+16
Fludioxonil	$C_{12}H_6F_2N_2O_2$	-33.25609	1.56732E+17
Linuron	$C_9H_{10}Cl_2N_2O_2$	-25.86655	4.10999E+14
Prometryn	$C_{10}H_{19}N_5S$	-18.94073	9.46179E+14
Propyzamide	$C_{12}H_{11}Cl_2NO$	-26.49343	4.40526E+15
Thiamethoxam	$C_8H_{10}ClN_5O_3S$	-29.34232	4.90090E+15

Table S6 DLPNO-CCSD(T)/aug-cc-pVTZ-PP corrections to the B3LYP/6-31+G(d) calculated adduct formation enthalpies. Negative corrections indicate lower formation enthalpies than those predicted by the B3LYP/6-31+G(d) method.

Molecule	Correction to formation enthalpy (kcal/mol)
Linuron	-2.2
Propyzamide	-2.1
Flusioxonil	0.3
Prometryn	-2.6
Thiamethoxan	0.8

Watermelon extract positive mode:

Protonated dimethomorph:

(a)

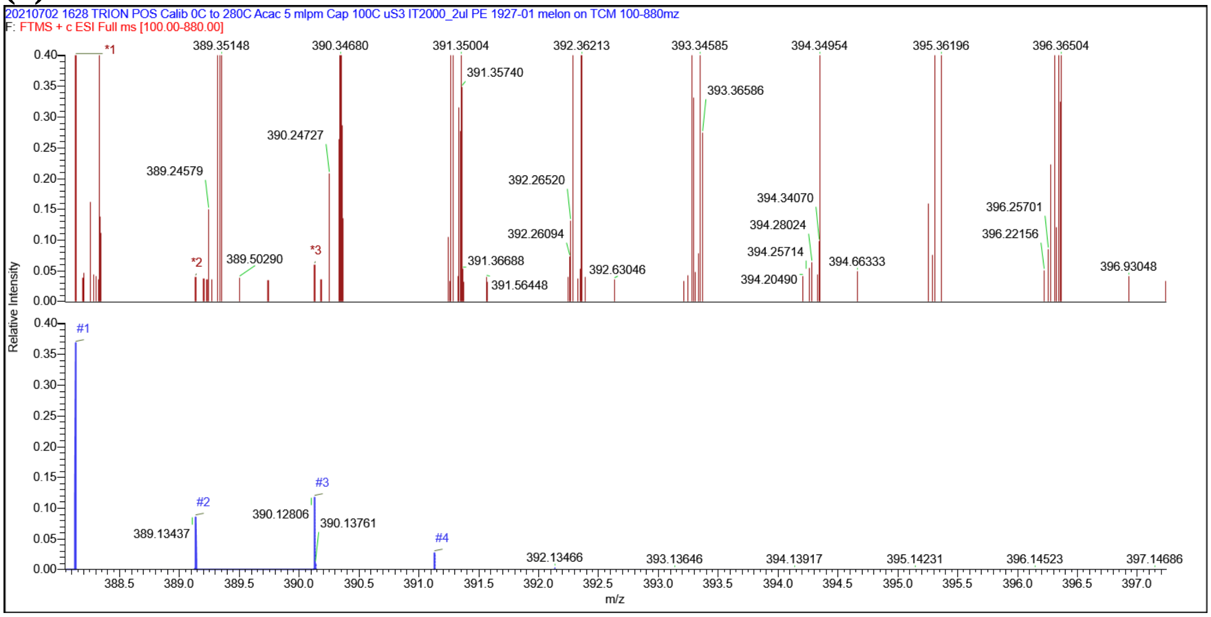

(b)

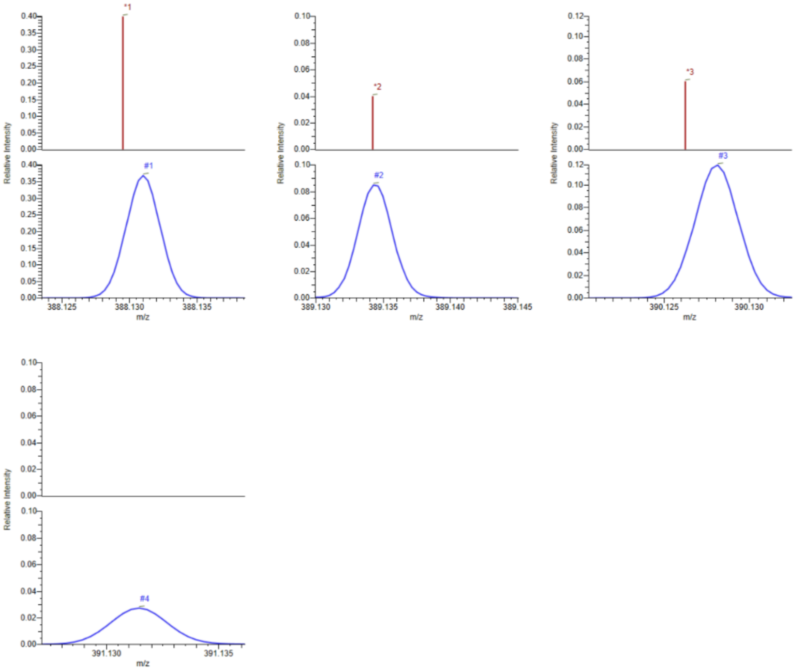

Figure S2 Isotope spectra of protonated dimethomorph in watermelon extract.
The depicted spectra illustrate the isotopes of protonated dimethomorph detected in the watermelon extract. The expected isotope peaks are in blue, while the detected isotope peak is marked in red.
(a) showcases the overall isotope spectra, while (b) provides a zoomed-in view of each individual peak for closer examination.

Watermelon extract positive mode:

Protonated imazalil:

(a)

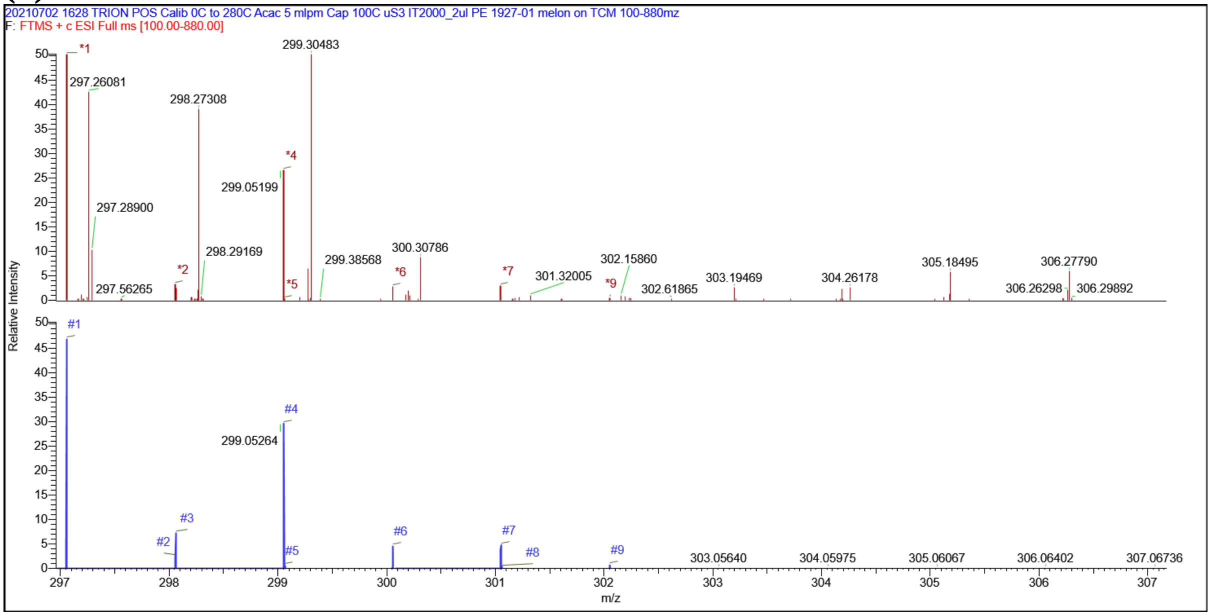

(b)

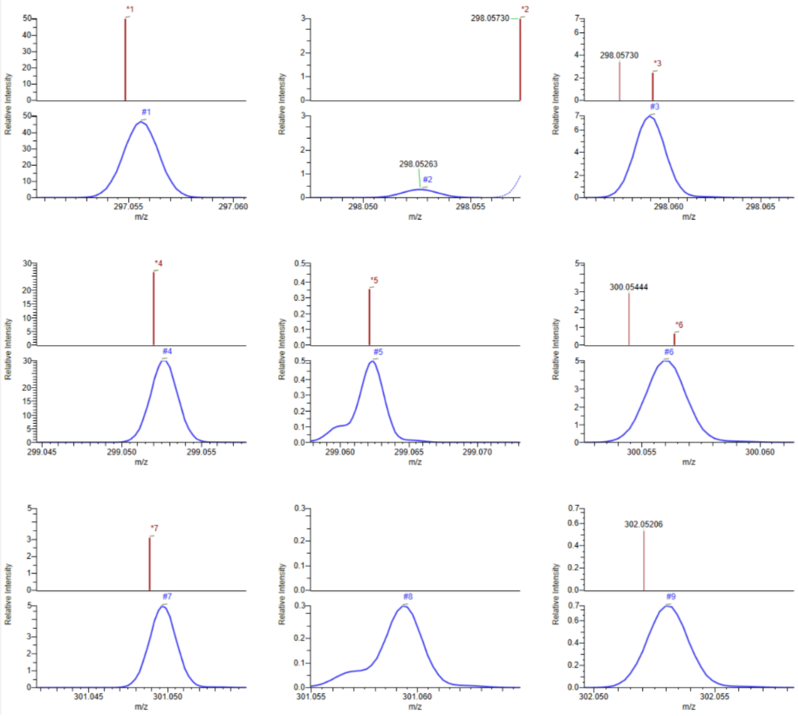

Figure S3 Isotope Spectra of protonated imazalil in watermelon extract
The depicted spectra illustrate the isotopes of protonated imazalil detected in the watermelon extract. The expected isotope peaks are in blue, while the detected isotope peaks are in red.
(a) showcases the overall isotope spectra, while (b) provides a zoomed-in view of each individual peak for closer examination.

Watermelon extract negative mode:

Dinotefuran bromide adduct:

(a)

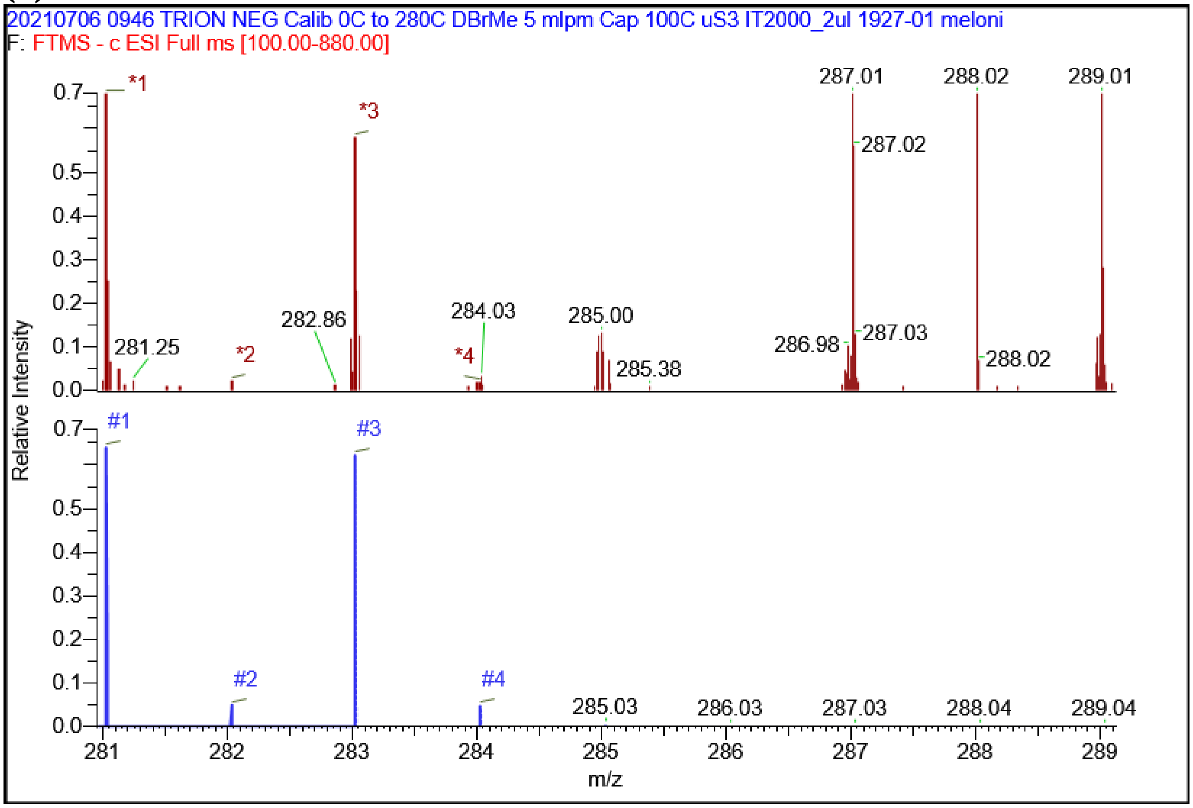

(b)

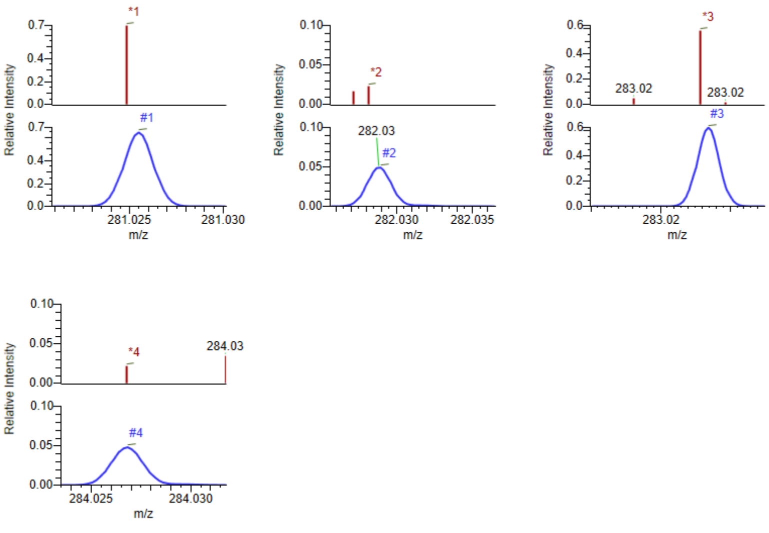

Figure S4 Isotope Spectra of dinotefuran bromide adduct in watermelon extract. The depicted spectra illustrate the isotopes of dinotefuran bromide adduct detected in the watermelon extract. The expected isotope peaks are in blue, while the detected isotope peaks are in red. (a) showcases the overall isotope spectra, while (b) provides a zoomed-in view of each individual peak for closer examination.

Lime extract positive mode:

Protonated azoxystrobin:

(a)

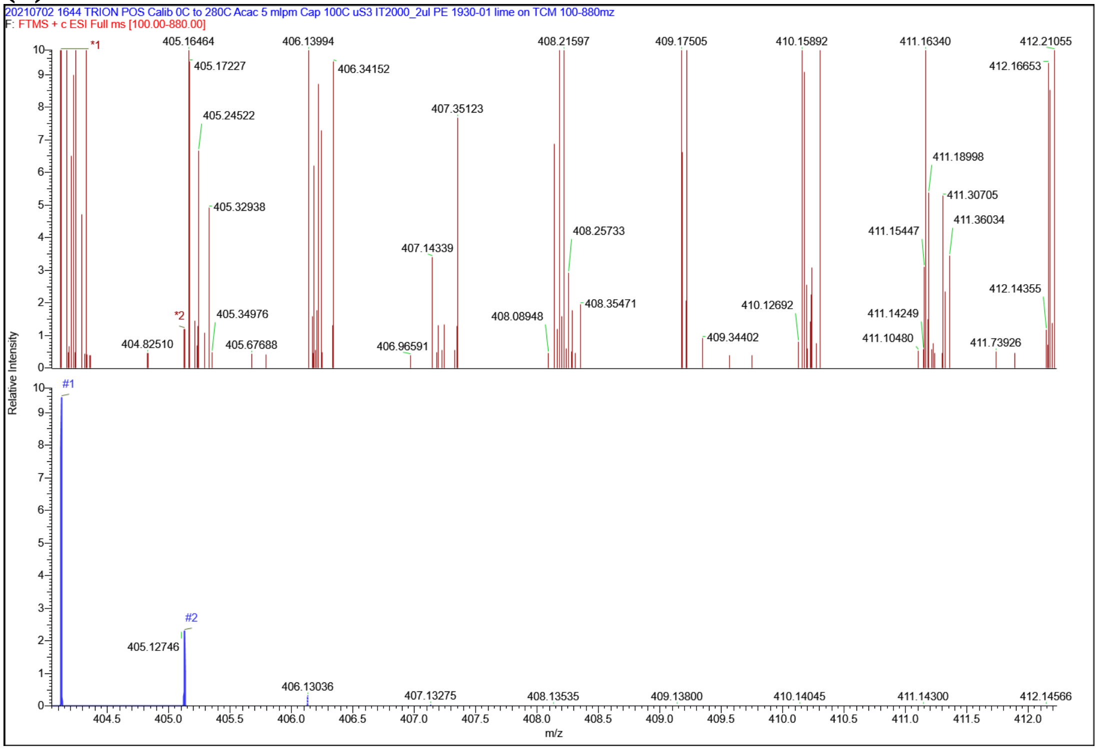

(b)

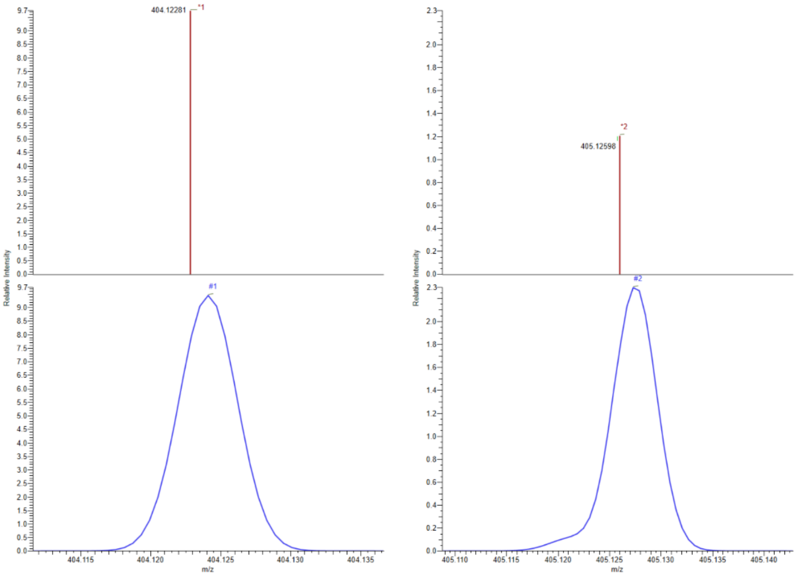

Figure S5 Isotope spectra of protonated azoxystrobin in lime extract.
The depicted spectra illustrate the isotopes of protonated azoxystrobin detected in the lime extract. The expected isotope peaks are in blue, while the detected isotope peaks are in red.
(a) showcases the overall isotope spectra, while (b) provides a zoomed-in view of each individual peak for closer examination.

Lime extract positive mode:

Protonated imazalil:

(a)

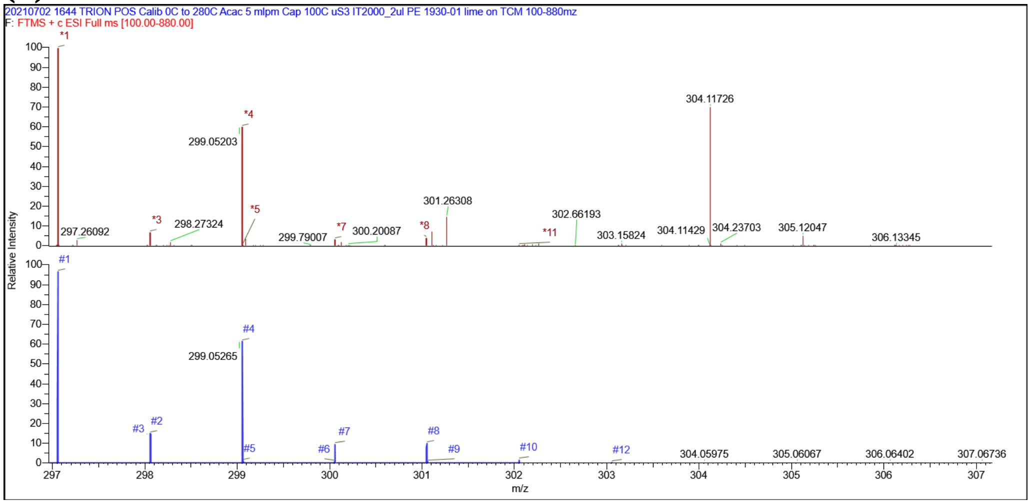

(b)

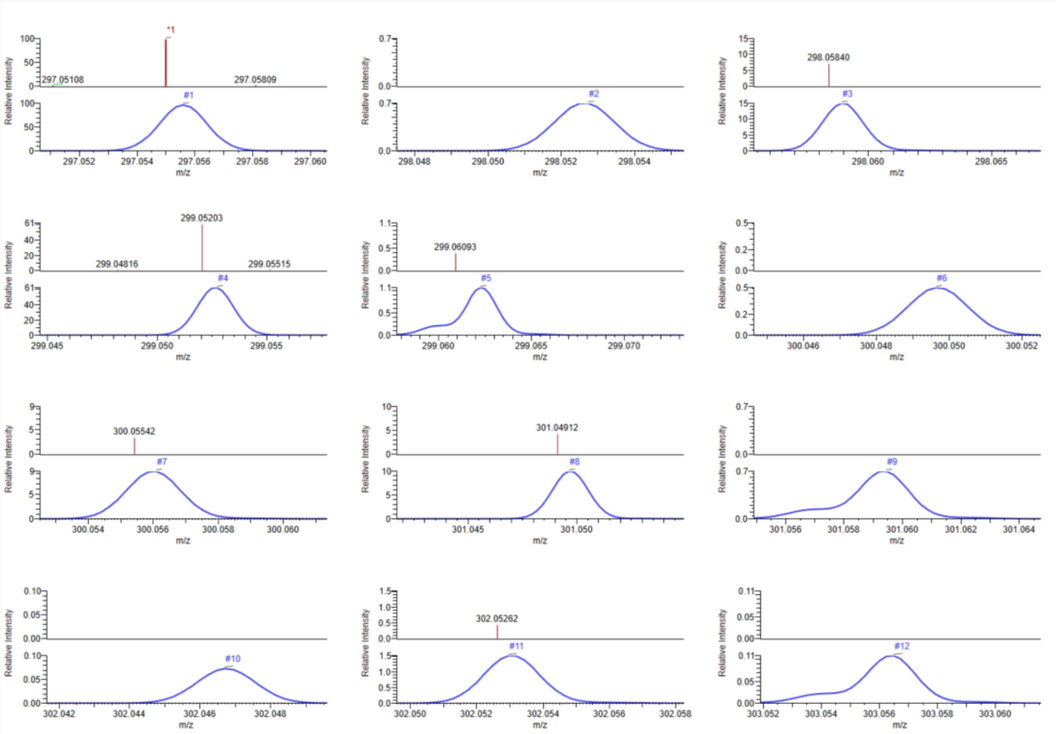

Figure S6 Isotope spectra of protonated imazalil in lime extract.
The depicted spectra illustrate the isotopes of protonated imazalil detected in the lime extract. The expected isotope peaks are in blue, while the detected isotope peaks are in red.
(a) showcases the overall isotope spectra, while (b) provides a zoomed-in view of each individual peak for closer examination.

Avocado extract positive mode:

Protonated thiabendazole:

(a)

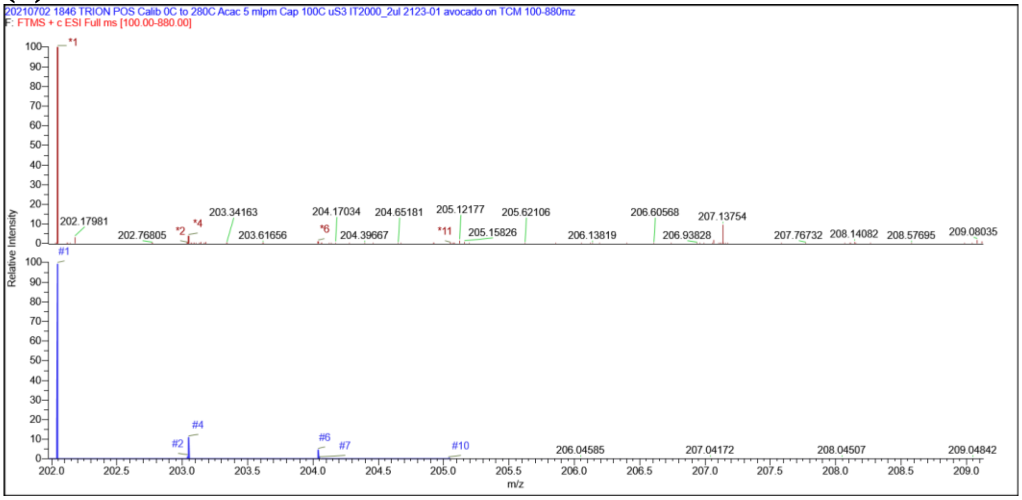

(b)

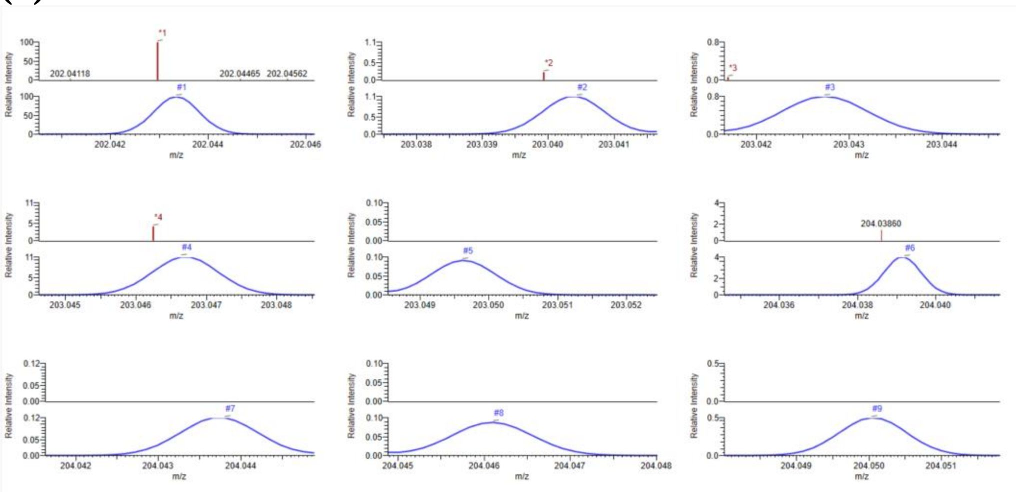

Figure S7 Isotope spectra of protonated thiabendazole in avocado extract.
The depicted spectra illustrate the isotopes of protonated thiabendazole detected in the avocado extract. The expected isotope peaks are in blue, while the detected isotope peaks are in red.
(a) showcases the overall isotope spectra, while (b) provides a zoomed-in view of each individual peak for closer examination.

Pineapple extract positive mode:

Protonated diazinon:

(a)

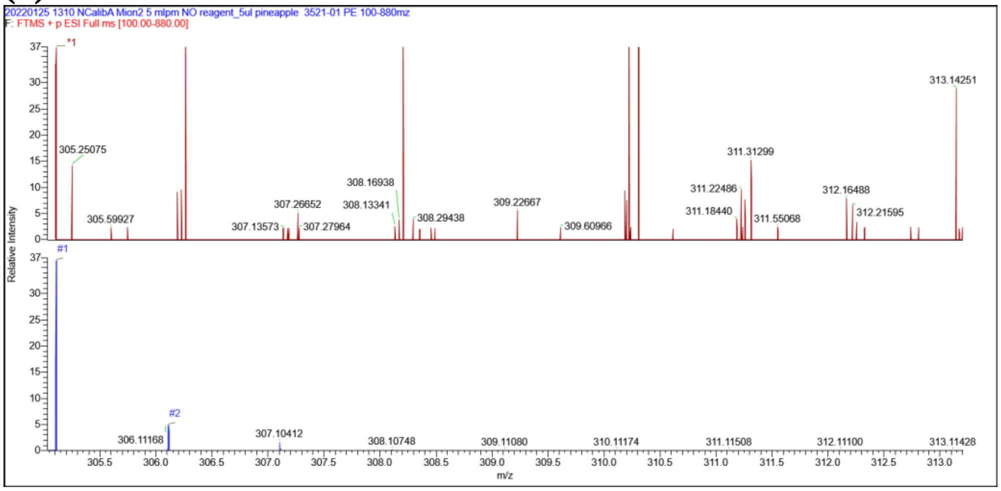

(b)

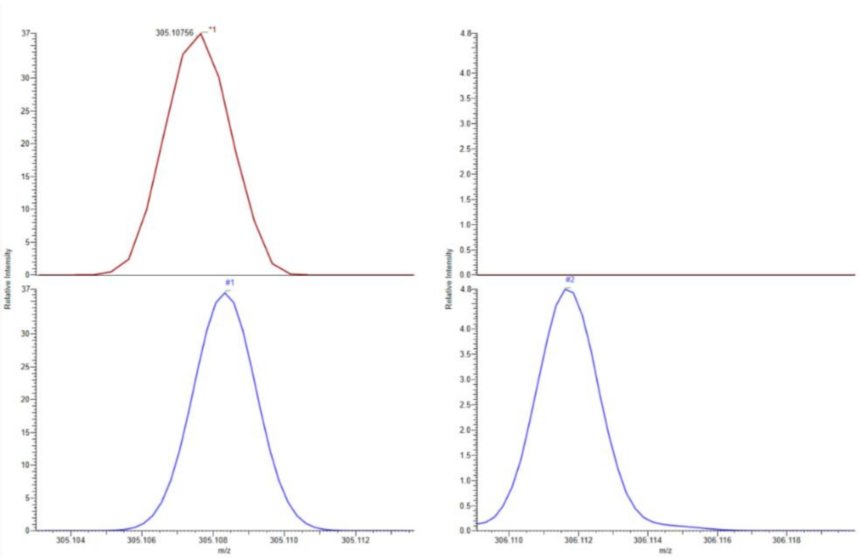

Figure S8 Isotope spectra of protonated diazinon in pineapple extract.

The depicted spectra illustrate the isotopes of protonated diazinon detected in the pineapple extract. The expected isotope peaks are in blue, while the detected isotope peaks are in red.

(a) showcases the overall isotope spectra, while (b) provides a zoomed-in view of each individual peak for closer examination.
